# The Double-Edged Sword: Conserved Functions of Extracellular Hsp90 in Wound Healing and Cancer

**DOI:** 10.3390/cancers6021065

**Published:** 2014-05-06

**Authors:** Michael W. Hance, Krystal D. Nolan, Jennifer S. Isaacs

**Affiliations:** Department of Cell and Molecular Pharmacology, Medical University of South Carolina, Hollings Cancer Center, Charleston, SC 29412, USA; E-Mails: hance@musc.edu (M.W.H.); dolek@musc.edu (K.D.N.)

**Keywords:** extracellular Hsp90, wound healing, cancer, motility, invasion, EMT, MMPs, inflammation, LRP1

## Abstract

Heat shock proteins (Hsps) represent a diverse group of chaperones that play a vital role in the protection of cells against numerous environmental stresses. Although our understanding of chaperone biology has deepened over the last decade, the “atypical” extracellular functions of Hsps have remained somewhat enigmatic and comparatively understudied. The heat shock protein 90 (Hsp90) chaperone is a prototypic model for an Hsp family member exhibiting a duality of intracellular and extracellular functions. Intracellular Hsp90 is best known as a master regulator of protein folding. Cancers are particularly adept at exploiting this function of Hsp90, providing the impetus for the robust clinical development of small molecule Hsp90 inhibitors. However, in addition to its maintenance of protein homeostasis, Hsp90 has also been identified as an extracellular protein. Although early reports ascribed immunoregulatory functions to extracellular Hsp90 (eHsp90), recent studies have illuminated expanded functions for eHsp90 in wound healing and cancer. While the intended physiological role of eHsp90 remains enigmatic, its evolutionarily conserved functions in wound healing are easily co-opted during malignancy, a pathology sharing many properties of wounded tissue. This review will highlight the emerging functions of eHsp90 and shed light on its seemingly dichotomous roles as a benevolent facilitator of wound healing and as a sinister effector of tumor progression.

## 1. Introduction

Cellular chaperones are essential for maintaining proteostasis, the balance between protein folding and degradation. Within this family of guardians, the abundantly expressed Hsp90 plays a critical protective role in countering protein misfolding and aggregation [1,2]. Hsp90 associates with a defined cohort of co-chaperones in an ATP-dependent manner to allow for precise regulation of its respective “client” proteins [3]. The advent of Hsp90 inhibitors has accelerated the discovery of hundreds of Hsp90 clients [4,5]. Although cytosolic protein kinases represent the predominant subclass of Hsp90 clients [6], many of which are implicated in malignancy [7,8,9,10,11], nuclear clients have also been characterized [12,13]. While the clinical utility of Hsp90 inhibitors has predominantly focused upon the targeting of malignant pathologies [3,14,15,16], the potential utility of these agents in protein folding pathologies such as neurodegenerative diseases has recently been reported [17]. Despite thousands of publications pertaining to cytosolic Hsp90 chaperone functions, it was only recently discovered that Hsp90 is also localized in mitochondria. This new locale for Hsp90 is predominantly observed in cancers, where it plays a critical role in regulating clients that oversee mitochondrial homeostasis and survival [18,19]. Taken together, a more comprehensive picture emerges, wherein Hsp90 orchestrates its protective effects and supports malignancy via regulation of numerous clients residing in multiple cellular compartments. Given the continuing evolution of Hsp90’s diverse functions, it should come as no surprise that Hsp90 also possesses unique extracellular functions. 

## 2. Out of the Box—Hsp90 on the Loose

### 2.1. Setting the Stage: Extracellular Chaperones

Nature provides us with continual surprises, including the discovery that a panoply of proteins demonstrate unexpected extracellular locations [20]. This phenomenon may enable cells to rapidly and efficiently respond to environmental cues and cellular stress. It is therefore a logical extension that several of the cytoprotective Hsp chaperones share this property of extracellular localization and function [21,22,23]. This geographic promiscuity endows chaperones with the ability to multi-task and broadens their sphere of biological function. To illustrate these trends, we will briefly highlight extracellular functions for the cytosolic chaperone Hsp70, its ER-resident paralog GRP78 (also known as BiP), and GRP94, the ER-resident paralog of Hsp90 (also known as gp96). 

### 2.2. Extracellular Chaperones Exhibit Immunoregulatory Functions

The cytoprotective functions of chaperones are often subverted within the cancer context, a trend exemplified by GRP78 and GRP94, which support cancer cell survival, progression, and therapeutic resistance [24,25,26]. Similar pro-tumorigenic functions have been reported for cytosolic Hsp70 [27,28,29]. Interestingly, the extracellular counterparts of these chaperones exhibit intrinsic tumor-repressive and tumor-supportive functions. The pioneering studies of Srivastava and colleagues initially ascribed tumor-specific antigenicity to these surface-localized chaperones [30,31], a function associated with their ability to chaperone antigenic peptides and to activate anti-tumor innate immunity [32,33,34,35,36,37,38]. Surface Hsp70 has been shown to initiate antitumor T cell responses via cross presentation of its chaperoned peptides to MHC molecules [27,39,40,41]. 

Despite this tumor-alerting role, the surface localization of Hsps may not be entirely beneficial to the host. A variety of stresses commonly found in solid tumors, such as ER stress and hypoxia, may stimulate the extracellular localization of these conventionally intracellular chaperones [42,43,44]. Not surprisingly, their extracellular localization is preferentially associated with the malignant phenotype [45,46,47,48,49,50,51,52,53]. Moreover, beyond chaperoning tumor antigens, surface GRP78 functions as a multifunctional receptor to execute myriad signaling events impacting cellular proliferation and survival [21,24,52,54,55,56,57]. These surface-localized chaperones have also been shown to function as ligands in tandem with surface receptors to trigger signaling events and cytokine release [21,40,58,59] which may support inflammation-associated tumorigenesis [60]. Finally, both GRP78 and Hsp70 may be secreted from tumor cells, with functional roles in chemoprotection, inflammatory signaling, and cell invasiveness [21,24,38,53,57,61,62,63,64]. Thus, these surface and secreted proteins exhibit functional diversity, adopting roles as chaperones, receptors or signaling mediators to modulate host immunity and tumorigenic responses.

### 2.3. Immunomodulatory Functions for eHsp90

Extracellular Hsp90 also possesses tumor-repressive and tumor-supportive regulatory functions. The first sighting of eHsp90 was reported in 1986, wherein Hsp90α and Hsp90β were identified as tumor antigens in chemically induced mouse tumors [65]. This Hsp90 “antigen” conferred anti-tumor immunity to subsequent tumor challenge in immunized mice [31,65]. Mechanistically, surface eHsp90 may participate in the cross-presentation of antigenic peptides, with the capacity to regulate both innate and adaptive immunity [33,66,67,68,69]. Although the multifunctional LDL receptor-related protein 1 (LRP1), also known as CD91, was identified as the common receptor for the extracellular chaperones Hsp90, GRP94 and Hsp70 and [70,71], eHsp90-mediated cross presentation may also be regulated by its interaction with the scavenger receptor SREC1 [68], indicating its capacity to partner with additional molecules. Similar to the complexity observed with other extracellular chaperones, eHsp90 also exhibits functional diversity in adopting chaperone-dependent [64,72] and chaperone-independent functions [73,74,75,76]. As will become evident, eHsp90 partners with a growing list of receptors and adaptors to elicit pleiotropic signaling events. Taken together, these reports indicate that eHsp90 may have been designed as a powerful danger signal to elicit potent protective immune responses against infection and cellular stress. Although these early reports of tumor eHsp90 highlight its anti-tumorigenic immunogenic function, we, and others have demonstrated pro-tumorigenic functions for eHsp90, a topic that will form the basis for the remainder of this review.

## 3. eHsp90 as a Facilitator of Wound Healing

### 3.1. Stress Promotes Hsp90’s Extracellular Location

Considerable controversy exists regarding the mechanism for Hsp90’s transit to the extracellular space, all the more puzzling given the apparent lack of a signal peptide to direct its extracellular localization. Early reports documented the release of Hsp90 and additional chaperones following necrotic death [77]. Less profound breaches of membrane integrity were also shown to promote Hsp90 secretion [65], implicating a regulatory mechanism for release. Subsequently, mechanisms for regulated secretion have been demonstrated, such as exosomal release [78,79,80,81,82]. A variety of stimuli are capable of inducing Hsp90’s extracellular localization including DNA damage [79], oxidative stress [83], chemotherapeutic agents [84,85], growth factors and signaling mechanisms [80,86,87,88], heat stress [78] and hypoxia [82,89], as recently reviewed [90]. However, it is presently unclear whether these stimuli similarly invoke an exosomal mechanism for Hsp90 secretion. It is interesting to note that a majority of these stimuli are linked with cellular stress and likely to be present in a wounded environment, supporting the notion that eHsp90 functions in a protective capacity to buffer cellular stress. Therefore, eHsp90 has seemingly taken a page from the intracellular Hsp90 playbook by functioning as a guardian of extracellular homeostasis. 

### 3.2. eHsp90 Is a Major Effector of Cell Motility

Although the intended physiological function of Hsp90 remains unclear, its adept response to cellular stress implies a conserved role in countering pathological conditions. Compatible with this notion, eHsp90 has been characterized as an essential mediator of tissue repair. Patsavoudi’s group was the first to demonstrate this novel function for eHsp90 over two decades ago. Thomaidou *et al*. [91] utilized brain membrane fractions from developing rats to generate an antibody (4C5) against a cell surface antigen later revealed to be Hsp90α [92]. Intense immunoreactivity was also observed in Schwann cells following mechanical injury [93]. Importantly, these studies were the first to elucidate a role for eHsp90 in cell motility. Functional inhibition of surface Hsp90 via antibody blockade validated that eHsp90 played a major role in Schwann cell migration [92,94], significant given that Schwann cell motility is an integral component of tissue repair following peripheral nerve injury [95]. Work from this group indicated that eHsp90 also possesses an intrinsic developmental role, supported by the robust detection of surface Hsp90 in embryonic and early postnatal neuronal tissues with a high propensity for neuronal migration. Moreover, antibody-mediated blockade of eHsp90 prevented the motility of cells associated with developing cerebellar explants of the central nervous system [92,94,96]. Another report demonstrated that exogenously added Hsp90 protein stimulated neurite formation [97]. More recently, eHsp90 has been implicated in development of the cranial mesenchyme during neurulation [98]. Taken together, these findings suggest a physiological role for eHsp90 in morphogenesis and wound repair, processes with a shared reliance upon cell movement and tissue regeneration.

**Figure 1:**
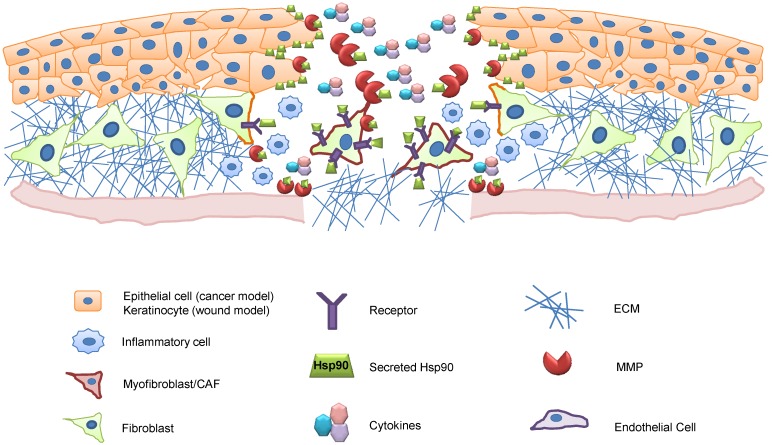
Role of eHsp90 in wound healing. Schema depicts a dermal wound, with the upper layer representing epidermal keratinocytes. Wounded keratinocytes demarking the wound edges secrete Hsp90 (green rectangles). Surface-localized Hsp90 initiates signaling events that promote cell motility into the wound bed. Surface and secreted eHsp90 also activate MMPs to facilitate wound closure, cytokine release, and vascular repair. In tandem, eHsp90-dependent signaling and eHsp90-activated MMPs collaborate to activate fibroblasts to a myofibroblastic-like state (depicted in red). Myofibroblasts are prevalent in close proximity to the wound site, whereas their activation diminishes at more distal sites, concomitant with reduced concentrations of eHsp90. Myofibroblasts play a key role in synthesizing the provisional matrix and creating an inflammatory milieu that is also important for host defense. Key for cell types is shown below.

Dermal models of injury have revealed a conserved pro-motility role for eHsp90 in wound healing, as depicted (Figure 1). Key events associated with dermal injury include the mobilization of dermal fibroblasts and keratinocyte migration to promote re-epithelialization [99]. Li and Woodley demonstrated that eHsp90 was required for hypoxia-mediated migration of dermal fibroblasts [89] and keratinocytes [100] and for TGFα-mediated migration of keratinocytes [80]. This wound repair activity of eHsp90 was further validated by the ability of topically applied Hsp90 protein to accelerate murine skin wound closure and re-epithelialization *in vivo* [89]. It was further demonstrated that eHsp90’s role in skin cell migration was dependent upon expression of the extracellular chaperone receptor LRP1 [80]. An eHsp90-LRP1 signaling axis similarly participates in hypoxia-dependent motility of skin cells [100]. The coupling of eHsp90 and LRP1 is an interesting partnership, as LRP1 is emerging as a key regulator of tissue damage and repair. LRP1 is upregulated during neural injury [101] and plays an important role in Schwann cell migration [102] as well as in inflammation and wound repair [103]. LRP1 has dozens of ligands [104], several of which are involved in pro-survival signaling during injury [105]. We, and others have shown that hypoxia upregulates LRP1 expression [10,106,107,108], and cell surface localization [10]. Thus, cellular hypoxia is a stimulus capable of facilitating both LRP1 surface expression and Hsp90 secretion, events expected to cooperate and amplify the eHsp90-LRP1 signaling axis under pathological conditions.

### 3.3. Evidence for eHsp90 in Matrix Remodeling

Although cell motility is important for wound repair, additional processes, such as wound contraction and matrix deposition, are essential components of the repair process. Modified fibroblasts, or myofibroblasts, at the injury site play a major role in these aspects of tissue repair [109]. A number of stimuli, such as TGFβ and mechanical stress, are well documented inducers of the myofibroblastic phenotype [110,111]. Myofibroblasts are characterized by expression of smooth muscle actin (SMA) and the appearance of SMA-containing stress fibers, cooperating events required for contractile force generation and wound closure [112]. Myofibroblasts actively participate in connective tissue remodeling via their expression and deposition of extracellular matrix (ECM) proteins such as vimentin, fibronectin, and collagen for the provisional matrix. Matrix remodeling is also achieved via the concerted actions of proteolytic enzymes, such as matrix metalloproteinases (MMPs). Importantly, MMPs have been implicated in keratinocyte migration and wound contraction [113], and as will be further elaborated, eHsp90 is a major regulator of MMP expression and activity in diverse cell types [73,114,115].

Our recent demonstration that eHsp90 contributes to formation of myofibroblasts [76] lends further support to the notion that eHsp90 modulates matrix remodeling. Importantly, MMP activity was essential for several eHsp90-initiated myofibroblastic properties, and the function and/or activity of a subset of MMPs were under eHsp90’s control. We demonstrated that eHsp90 regulates MMP-3 expression in eHsp90-initiated myofibroblastic cells [76]. Interestingly, MMP-3 is an important facilitator of the myofibroblastic phenotype [116], and eHsp90 was recently demonstrated to regulate MMP-3 activity during morphogenesis [117]. Thus, the ability of eHsp90 to promote a myofibroblastic phenotype adds mechanistic insights into eHsp90’s complex roles in tissue repair. The ability of eHsp90 to orchestrate multiple components of the repair process, including myofibrobast generation, is undoubtedly a critical component of eHsp90’s stimulation of *in vivo* epithelialization. 

## 4. The Dark Side: eHsp90’s Linkage with Malignancy

### 4.1. Sightings of eHsp90 in Cancer

Over the past decade, a more sinister side of eHsp90 has emerged, revealing a role for eHsp90 in malignancy. Whereas earlier studies characterized tumor eHsp90 as a protein capable of alerting the host immune system to danger [65], an avalanche of subsequent findings have supported the notion that eHsp90 promotes tumor progression. Further evidence of eHsp90’s widespread role in tumorigenesis is provided by its frequent detection on the surface of diverse tumors including fibrosarcoma [114], breast [87], melanoma [118], ovarian [119] and neuroblastoma [120]. Studies from our group have demonstrated surface Hsp90 in tumor cells from both glioblastoma multiforme (GBM) and prostate cancer [10,121]. One pressing question is whether the *in vitro* identification of tumor surface Hsp90 has clinical relevance. In response to this valid query, Hsp90 has been detected on the surface of primary melanoma cells and resultant metastases [122]. Moreover, we have detected Hsp90 on the surface of primary prostate cancer specimens, and further identified the selective expression of transcripts associated with enhanced tumor aggressiveness in tumor cells with elevated levels of surface Hsp90 [121]. These latter studies provide compelling support for the notion that surface Hsp90 occurs within a clinical context. 

Tumor cells also secrete Hsp90, indicating that both surface-localized and secreted Hsp90 populations contribute to malignancy. Although environmental stress stimulates Hsp90 secretion, Hsp90 is also preferentially secreted from cancer cells in the absence of exogenous stress, as shown in melanoma [123], breast [87], and our aforementioned studies in GBM and prostate cancers [10,121]. Clinical relevance for tumor-directed Hsp90 secretion is supported by demonstrations of elevated Hsp90 in patient serum, exemplified by the elevated Hsp90 levels in serum or plasma in patients with prostate, liver, breast, lung, pancreatic, and hepatocellular tumors relative to cancer-free controls [87,124,125]. Of particular interest, patients with metastatic disease exhibited the highest levels of serum Hsp90 [87,124]. The presence of Hsp90 autoantibodies in patients with late stage ovarian cancer [119,126], breast cancer [127], and osteosarcoma [128] lends further support to tumor-derived circulating Hsp90. Although the functional relevance of secreted Hsp90 is not clear, a recent study demonstrated that a subset of colorectal cancer patients with elevated serum Hsp90 exhibited increased expression of integrin alphaV, a potential target of eHsp90, in the corresponding primary tumors, [129]. Further evidence for a functional role for secreted Hsp90 is demonstrated in that exosomal Hsp90 is responsible for a significant proportion of the pro-invasive activity of these secretory vesicles [81,130]. Finally, increased expression of rab27B, which regulates the exosomal release of Hsp90, is associated with breast cancer lymph node metastasis [130].

### 4.2. Role of eHsp90 in Cancer Cell Motility, Invasion and Metastasis

The widespread expression of eHsp90 in diverse cancers portends an important function. In keeping with this prediction, eHsp90 has emerged as a pivotal regulator of tumor cell motility, invasion and metastasis [131,132]. The first validation of eHsp90’s role in cancer cell motility and invasion was demonstrated by functional blockade of eHsp90, wherein cancer cells treated with either anti-Hsp90 antibody or with derivatized cell impermeant small molecule Hsp90 inhibitors effectively suppressed tumor cell motility and invasion [92,114,118]. In recent years, a flurry of papers has reinforced the pro-motility and/or pro-invasive factor functions of eHsp90 in cancer [73,84,87,133,134] including our work in GBM and prostate cancer [10,121]. A number of studies have evaluated the effects of eHsp90 blockade *in vivo*. While one study demonstrated that this approach suppressed primary tumor growth [73], other studies reported no such effects on the primary tumor [87,118] However, all studies unanimously support the conclusion that blockade of eHsp90 function profoundly impairs tumor invasion and metastasis [10,73,87,118,135], findings that reinforce this fundamentally conserved role for eHsp90 in cancers. 

### 4.3. eHsp90 Regulates MMP Activity

Tumor invasion is strongly implicated in tumor intravasation and extravasation, which represent important components of the metastatic cascade. Given that tumor metastasis causes the majority of cancer-related deaths [136], an understanding of how eHsp90 may regulate these processes has potentially high clinical relevance. Although several pathways are implicated in tumor invasion, activation of the MMP enzymes represents a critical proteolytic hub regulating cancer invasion and progression [137,138]. Interestingly, a number of reports identify eHsp90 as a major regulator of MMP activity. In fact, Hsp90α was detected in a regulatory complex with MMP-2 in one of the first reports illustrating the pro-invasive function of eHsp90 [114]. Subsequently, eHsp90α was found to regulate MMP-2 stability and/or activity in additional models [73,87]. eHsp90 has also been reported to regulate MMP-9 activity and tumor invasion as a component of an extracellular complex with the hyaluronan receptor CD44 [134]. Although the majority of reports implicate Hsp90α as an MMP-interacting pro-motility factor, other reports indicate that both isoforms may interact with MMP-2 and MMP-9 [115]. This interchangeability is not surprising given the high homology between the isoforms [139]. Some of this variability may also be explained by the various isoform-specific antibodies used to interrogate Hsp90 function, coupled with potential alterations in eHsp90’s tertiary structure and/or interacting proteins that may sterically hinder antibody access. Recent reports indicate that eHsp90 may have a role in the activation of additional cell surface proteases involved in tumor cell motility [81]. Thus, although possible that Hsp90α and Hsp90β may possess distinct functions within some contexts, eHsp90’s modulation of MMP activity is emerging as a powerful executor eHsp90’s invasive functions.

### 4.4. eHsp90 Regulates Receptor Signaling

Extracellular Hsp90 cooperates with a growing number of transmembrane receptors to modulate signal transduction. Not surprisingly, eHsp90 partners with its immunomodulatory receptor LRP1 to drive cell motility in a number of cancers, as shown in colon cancer [129]. Our studies support conservation of an eHsp90-LRP1 pro-motility pathway in prostate and GBM [10,121]. In addition to LRP1, eHsp90 activates EGFR signaling in breast cancer, as shown by its interaction with the extracellular domain of HER2/Neu/ErbB2 [133], the ligandless co-receptor for EGFR family members [140]. This interaction was a requisite for ligand-mediated EGFR3/HER2 dimerization, signal transduction and invasive activity [133]. Another report demonstrated that eHsp90 facilitated EGFR endocytosis and stimulated receptor activity and cell migration in GBM [141]. Interestingly, this pathway required TLR4 activity, receptors that normally function as innate receptors critical for host defense. Our recent studies also support potential functional cooperativity between eHsp90 and TLR4 [142]. At least two studies have demonstrated that eHsp90 regulates integrin signaling, either by influencing its interaction with downstream intermediates [118] or by regulating integrin expression [129]. Finally, we demonstrated that eHsp90 was essential for regulating ligand-independent EphA2 activation and subsequent glioma invasion [10]. In addition to serving as a signaling conduit for eHsp90, several reports indicate that a subset of transmembrane receptors may serve a dual purpose in tethering eHsp90 to the cell surface. In support of this, cell surface CD44 was required for surface expression of Hsp90 [134]. Similarly, we observed that LRP1 was essential for the surface expression of LRP1 in GBM [10]. 

**Figure 2:**
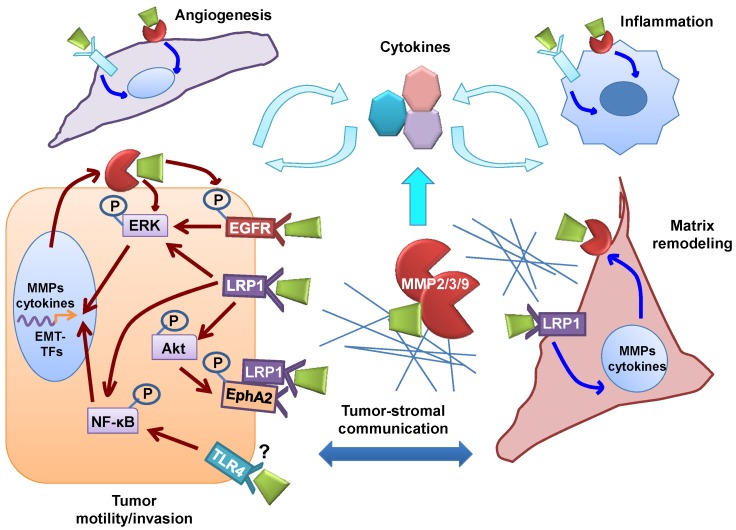
Role of eHsp90 in cancer. (Refer to cell type key in Figure 2). Summary of eHsp90 functions compiled from published reports in cancer epithelial and glioma cells (depicted by red arrows). eHsp90 interacts with a number of receptors (not directly confirmed for TLR4) to initiate signaling events converging upon AKT, ERK and NF-κB to facilitate cell motility. eHsp90-mediated activation of ERK and NF-κB promote epithelial to mesenchymal transition (EMT) activation in prostate and colon cancer, respectively. EMT activation collaborates with eHsp90-dependent signaling to further stimulate expression of MMPs and cytokines. Surface eHsp90 may also activate ERK through a variety of signaling intermediates. Surface eHsp90 signaling and MMP activation also elicits a CAF-like stromal reaction and promotes neoangiogenesis. CAF-like cells contribute to the inflammatory milieu, to the desmosplastic stroma, and to the angiogenic response. The earliest reports identified surface Hsp90 on inflammatory cells, thereby also implicating eHsp90 as a direct modulator of immune components in the cancer microenvironment. Finally, eHsp90 has been shown to interact directly with matrix components, indicating an accessory role in ECM remodeling. These collective events create an intricate web of cancer-stromal interactions that have the potential to create robust feed-forward networks that drive cancer progression.

In addition to these direct mechanisms, a number of key intracellular signaling intermediates participate in eHsp90’s pro-motility and/or invasive functions such as Src [118,141], PKCδ [141], NF-κB [129,141], and ERK [121]. The ability of signaling intermediates to form feed-forward circuits significantly increases pathway complexity. For example, eHsp90-mediated Src activation may potentiate receptor activation, as observed with integrins [118] and EGFR [141]. Compatible with this notion, we demonstrated that eHsp90-dependent Src activation in GBM facilitated AKT-mediated formation of a pro-invasive complex between EphA2 and LRP1 [10]. In addition, MMPs are well known effectors of cellular signaling [143] also capable of modulating eHsp90-directed signaling events, such as the liberation of growth factors to amplify eHsp90-activated receptor signaling [141]. Moreover, we have shown that MMP-2/9 is positioned both upstream and downstream of signaling intermediates such as ERK, indicating a complex bi-directional crosstalk mechanism [121]. Thus, a more comprehensive picture of eHsp90 signaling emerges, which includes eHsp90’s direct interaction with cell surface receptors and proteolytic enzymes, signal transmission to downstream intermediates, and crosstalk between these intermediates and additional receptors and adaptor molecules, as depicted (Figure 2).

## 5. Similarities between Wound Healing and Cancer

How is it possible to reconcile the seemingly disparate functions of eHsp90 as a beneficial facilitator of wound healing and as an effector of tumor progression? To understand this apparent dichotomy, it is important to appreciate the conserved pathways in wound healing and cancer. In 1858, Rudolph Virchow proposed his irritation theory for cancer. This astute observation was based upon that fact that neoplasms frequently developed at sites of chronic irritation. Subsequently, irritation or injury was recognized as a causative factor in cancer [144]. The pioneering studies of Mina Bissell validated this collaboration between injury and tumor progression [145]. The notion that chronic wounds and associated inflammation are risk factors for tumor promotion [146,147] is well established, with inflammation now recognized as a major hallmark of cancers [148]. 

A singular goal of repair following tissue injury is to achieve wound closure and barrier restoration. The wound response is characterized by a complex series of integrated and overlapping components including cell motility, cell proliferation, matrix deposition and remodeling, inflammation and neoangiogenesis [99,149,150]. Cancers co-opt many effectors of the wound healing response, and features of a wound healing environment are commonly found in malignant and pre-malignant conditions [151,152,153]. Indeed, Dvorak is credited with the well known phrase that “cancers represent wounds that do not heal”. As excellent reviews exist on these comparisons, a subset of shared processes will be briefly highlighted and subsequently discussed within the context of eHsp90 functions.

### 5.1. Hypoxia and Angiogenesis

Hypoxia in wounded skin is frequently due to vascular disruption and the higher oxygen demands of regenerating tissue. While transient hypoxia is required for vascular regeneration and repair in wounding [154], persistent hypoxia in the tumor microenvironment (TME) supports vascular permeability, tumor progression and metastasis [155,156,157,158,159]. Hypoxia also elicits a potent proinflammatory response [160,161,162].

### 5.2. Inflammation

Inflammation serves a number of essential roles during tissue injury. Inflammation activates the host immune system to elicit a protective response to guard injured tissue from pathogens [163]. Inflammation is also critical for stimulating neoangiogenesis and for enhancing the recruitment of stromal cells critical for the regenerative response [164]. Although designed to elicit a transient protective response to injury, the inflammatory milieu that persists in cancer supports tumor growth, angiogenesis, and tumor progression [148,165,166,167]. The recruitment of inflammatory cells also serves as an important source of proteases that contribute to degradation and remodeling of the extracellular matrix.

### 5.3. Matrix Remodeling

In normal wound healing, matrix deposition and remodeling are essential components of repair. Inflammatory cytokines released from the injured tissue promote formation of modified fibroblasts, or myofibroblasts, which play an essential role in many phases of the repair process [99,168,169]. The extracellular milieu of a wound is highly proteolytic, and myofibroblasts are major effectors of connective tissue remodeling due to their synthesis and proteolytic degradation of extracellular matrix (ECM) components [112,170,171]. In the cancer microenvironment, these myofibroblastic cell types are termed cancer-associated fibroblasts (CAFs). Similar to their counterparts in wound repair, CAFs are main producers of the tumor-surrounding ECM [172] and are key instigators of formation of the desmoplastic stroma that characterizes many advanced carcinomas. Whereas myofibroblasts resolve following wound repair [173], the constant remodeling of the tumor stroma sustains the myofibroblast population [172], which further supports the inflammatory and angiogenic millieu [174,175,176,177]. The pivotal role of CAFs in tumor growth, invasion, and metastasis is well documented [175,178,179,180,181,182]. 

### 5.4. Cell Motility and Invasion

Cell motility and invasion are critical components of the wound repair response [99,149]. In dermal wounds, epithelial cells, fibroblasts, keratinocytes, and other stromal cell types migrate inward towards the wound bed to achieve barrier restoration, wherein the provisional matrix serves as a scaffold for the migrating cells. In tandem, proteolytic enzymes such as MMPs diminish cellular adhesion to the basal lamina to facilitate the cellular motility required for repair [99,183]. These physiological repair processes are efficiently co-opted in cancer. In malignancy, the aberrant matrix serves as a highway for tumor cell motility, cancer cells acquire motile and invasive properties, and the constitutive ECM remodeling enables tumor escape [148,184,185].

In summary, many of the normally reparative processes associated with wound healing are constitutively activated in the tumor milieu [153,186]. The reactive stroma typically observed in cancers mirrors the myofibroblastic wound healing response [187,188,189] and an inflammatory stroma has been clinically associated with invasive disease and outcome [190]. Moreover, a gene expression signature indicative of wounding is predictive for cancer progression in a wide range of malignancies [191], a trend especially noted in breast cancer [190,191].

## 6. eHsp90 Is a Critical Factor in Wound Healing and Cancer

A picture emerges wherein eHsp90 orchestrates a continuum of events that support physiological wound healing and malignancy. This section will highlight the known molecular mechanisms for eHsp90 function and illustrate the duality of these processes in wound healing and cancer. 

### 6.1. eHsp90 Regulates Angiogenesis

Hypoxia, a microenvironmental stimulus common to both wound healing and cancer, elicits Hsp90 secretion within both contexts [75,100]. Given that hypoxia in wound injury is designed to trigger vascular repair, it is not surprising that eHsp90, and in particular eHsp90α, affects a variety of cell types associated with vascular development. An early report indicated that eHsp90 modulated cellular signaling events in vascular smooth muscle cells (VSMCs) [83], while a subsequent report showed that eHsp90 promoted the motility of human microvascular endotheial cells (HMECs) and enhanced tubule formation [88]. Interestingly, this same group showed that a variety of growth factors, or the presence of ECM components, increased Hsp90 secretion from HMECs. This finding indicates that multiple stimuli coexisting within the wounded microenvironment stimulate reinforcing mechanisms to ensure Hsp90 secretion and subsequent repair of the damaged vasculature. To place this within a physiological context, Hsp90 was demonstrated to accumulate on the surface of regenerating tissue, and eHsp90 function was essential for neoangiogenesis in a mouse skin wound healing model [88]. Although the role of eHsp90 in the regulation of tumor vasculature is not well characterized, it was recently shown that eHsp90 regulates tumor angiogenesis *in vivo* [73]. Therefore, tumor secreted Hsp90 likely activates angiogenic pathways that are normally beneficial for vascular repair in wound healing. This notion is further supported by our prior studies demonstrating that in response to viral (pathogenic) challenge, eHsp90 exhibited a conserved ability to promote the secretion of angiogenic factors and sustain cell motility in dermal endothelial and cancer epithelial cells [192].

### 6.2. eHsp90 as a Pro-Inflammatory Factor

The earliest reports depicted eHsp90 as a protective mediator designed to alert the host to pathogenic stress [193]. Within this capacity, eHsp90 activates a number of inflammatory effectors and elicits the secretion of numerous inflammatory cytokines [66,77,194]. Recombinant Hsp90 induces the release of inflammatory cytokines in a wide range of epithelial and stromal cell types [76,77,192,194,195], indicating that cells have evolved conserved mechanisms to respond to eHsp90-mediated inflammatory pathways. By extension, eHsp90 utilizes similar conserved mechanisms to control inflammation in malignant tissues, with TLRs, NF-kB and LRP1 representing the key known pro-inflammatory eHsp90-regulated signaling nodes. TLRs play a major role in wound healing and cancer [196,197,198]. Of this family, eHsp90 appears to predominantly partner with the TLR4 receptor, as shown within the context of bacterial challenge [123,199]. NF-kB plays a central role in wound healing and cancer [147,163,196,200] and eHsp90 activates NF-kB in cancer cells [201]. We also reported that an eHsp90-NF-kB pathway is required for release of inflammatory cytokines in endothelial and prostate stromal cells [121,192], an activity consistent with its putative role in myofibroblast formation during the inflammatory phase of wound healing. The TLR4 and NF-kB pathways may collaborate in inflammation and cancer [202,203], and eHsp90 facilitates the secretion of inflammatory cytokines via a TLR4-NF-kB pathway in vascular cell types [195]. LRP1 is an acknowledged mediator of inflammation and wound repair, with additional roles in malignancy [103,204,205]. Although eHsp90-LRP1 regulated cytokines have not been well characterized, eHsp90 has been reported to signal through an LRP1-NF-kB pathway in cancer cells [201]. 

### 6.3. eHsp90 Regulates Matrix Remodeling

Matrix deposition and remodeling are essential components of wound repair. While this process subsides in wounding, the ECM is constantly remodeled in the tumor stroma, which resembles a chronically wounded reactive microenvironment. Fibronectin (FN) production is increased at healing wounds [206], and FN is also a major component of the cancer stroma [207,208], observations consistent with the notion that the tumor stroma shares many processes associated with wound healing. Recently, eHsp90 was shown to induce FN expression in breast and colon cancer cells [201,209]. Interestingly, eHsp90 associated with extracellular FN [209], indicating that eHsp90 may play a direct role in matrix assembly. ECM proteins including FN also stimulate Hsp90 secretion in endothelial cells [88], suggesting that a complex interplay between eHsp90 and the ECM is conserved in a number of cell types. ECM molecules play a protective role in buffering growth factors from degradation and providing biological latency [210]. Aberrant upregulation of proteolytic enzymes in the cancer stroma enhances the enzymatic degradation of basement membranes, which liberates bioactive molecules and correlates with metastatic potential [211]. MMPs, which are essential for wound healing, are also among the major regulators of this proteolytic turnover in malignancy [138,212].

Myofibroblasts and CAFs are vital to the ECM remodeling process in wound healing and cancer via their upregulation MMPs, including MMP-2, MMP-3 and MMP-9 [116,143,172,213,214]. Our findings indicate that eHsp90 may regulate MMP activity on several levels. First, eHsp90 initiates the formation of CAF-like cells [76], which are key culprits of the reactive stroma. Second, eHsp90 induces MMP-3 expression in CAF-like cells. Third, MMP activity is essential for manifestation of several CAF-specific properties, indicating that eHsp90-dependent MMP upregulation is also a key event for generation and/or maintenance of CAFs. Moreover, we, and others have shown that eHsp90 regulates the expression and activity of MMP-2 and MMP-9 in tumor cells [73,87,114,115,121,134]. Thus, eHsp90 regulates proteolytic functions of tumor and stromal cell types, a toxic combination when considered within the context of the consequences for matrix remodeling, tumorigenic signaling, and tumor-stromal communication [184,215] (as depicted in Figure 2). In addition to generating the tumor reactive stroma, eHsp90-regulated MMP activity enables destruction of the normal interstitial architecture, which is a major facilitator of the aforementioned tumor invasion and metastasis.

### 6.4. eHsp90 Regulates Cell Motility in Injury-Induced and Cancer Models

The eHsp90-dependent regulation of cell motility and invasion is by far its most prominent role, highlighted by its crucial function in models of injury-induced migration and cancer. Functional conservation is strongly supported by the shared molecular events participating within these contexts. For example, the eHsp90-LRP1 signaling axis is essential for dermal cell motility following injury [80,100] and eliciting cancer cell motility and invasion [10,121,129]. Moreover, AKT activation was recently shown to be a key effector for eHsp90’s migratory and *in vivo* healing functions [216]. By the same token, we identified an eHsp90-LRP1-AKT pathway as a primary regulator of GBM cell motility and invasion [10]. Our work further demonstrated that his eHsp90-LRP1-AKT pathway coupled to EphA2 receptor signaling, a pathway regulating the invasive activity of numerous cancers [217,218]. Although a direct eHsp90-EphA2 linkage has not yet been reported in wound repair, EphA2 and related family members are well known mediators of inflammation and regeneration [219,220]. LRP1 may also partner with the inflammatory mediator NF-kB to elicit eHsp90-mediated colon cancer cell motility [129], while NF-kB was shown to exert pro-motility effects via TLR4 in GBM [141]. These findings illustrate that a cohort of molecules with established inflammatory and/or or wound repair roles collaborate with eHsp90 to facilitate cell migration in a variety of cellular contexts. 

EGFR and ERK are also emerging as major effectors of eHsp90-mediated cell motility in wounding and cancer. EGF is released by injury [221] and activation of the EGFR pathway is a central feature of wound healing [99,168]. TGFα, a ligand for EGFR, was required for Hsp90-dependent keratinocyte migration [80]. EGFR signaling was also important for eHsp90’s pro-motility function in a number of cancer models [133,141]. ERK is essential for wound repair [222,223], and EGFR and ERK may participate in a signaling cascade [221,224]. We demonstrated that eHsp90-ERK signaling is required for the motility of both prostate stromal fibroblasts and prostate cancer cells [76,121]. In the latter instance, we showed that eHsp90-LRP1 dependent signaling was essential for ERK activation and for supporting eHsp90’s motogenic effects. Finally, we demonstrated that MMP activity represents a critical component of eHsp90’s ERK-dependent migratory activity. Collectively, these studies illustrate that a number of collaborative pathways reinforce and sustain the migratory functions of eHsp90 to modulate wound closure and cancer cell invasion. 

## 7. The Big Picture: eHsp90 Initiated EMT Integrates Wound Healing and Cancer Progression

### 7.1. eHsp90 Initiates the EMT Program

Cells participating in wound healing and cancer activate shared pathways to transition from a sedentary to a motile phenotype. Activation of the developmental genetic program epithelial to mesenchymal transition (EMT) represents one of the primary mechanisms orchestrating this behavioral change. Many excellent reviews have discussed the broad similarities between the developmental EMT required for physiological morphogenesis and the pathological EMT associated with cancer [185,225,226]. Reactivation of the EMT program in malignancy is associated with increased tumor invasion and is considered a primary culprit for metastasis and cancer associated lethality. Receiving somewhat less attention is the comparison between EMT and tissue regeneration associated with injury. The wound closure process utilizes a mechanism analogous to EMT, characterized by the acquisition of mesenchymal morphology and increased migratory potential [227]. 

**Figure 3:**
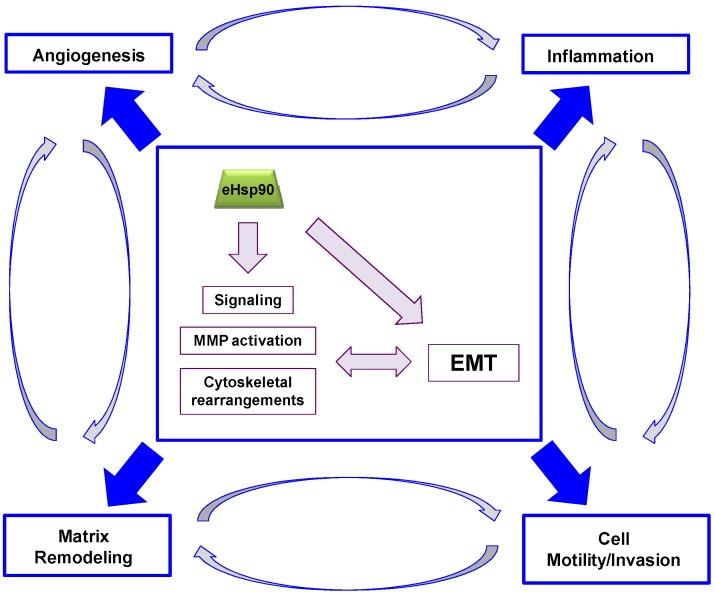
Conserved functions of eHsp90 in wound repair and cancer. Central box depicts key molecular aspects of eHsp90 action highlighted in this review. These molecular events have the potential to elicit or support EMT activation, and EMT activation is reciprocally associated with heightened activation of these functions. Some of the key functional outputs of eHsp90 function are depicted in each corner of the box: Angiogenesis, Inflammation, Matrix Remodeling and Cell Motility and Invasion. As indicated, each of these functions may crosstalk and integrate with any of the other outputs to contribute to the highly complex tumor microenvironment.

The full molecular reprogramming occurring during an EMT is primarily orchestrated by three major groups of transcription factors: the ZEB, Snail and Twist families. [228,229,230]. Reactivation of these EMT transcription factors (EMT-TFs) in cancer is a crucial step in initiation of the invasion-metastasis cascade. These EMT-TFs also play crucial roles in wound repair [227]. Remarkably, eHsp90 is emerging as a main orchestrator of the EMT program. We recently showed that eHsp90 is capable of inducing the transcription and expression of members from each of the 3 primary EMT-TF families in prostate cancer cells [121]. Shortly thereafter, eHsp90 was demonstrated to elicit an EMT response in colon cancer cells [201]. Thus, the ability of eHsp90 to direct EMT events considerably broadens our understanding and appreciation of its conserved functions in wound repair and cancer. We have herein highlighted eHsp90’s central command of MMP activity and signal transduction, with consequent effects upon processes highly pertinent to wound repair and cancer, including angiogenesis, inflammation, matrix remodeling and cell motility, events inextricably linked with EMT activation in cancer and wound healing [148,227]. Within this context, we will revisit the known effectors of eHsp90 action within the broader context of the EMT program. A general schema reinforcing these trends is shown (Figure 3).

### 7.2. eHsp90’s Pro-Motility Effectors are also Drivers of EMT

Increased cell motility is a well known hallmark of EMT, and a striking picture emerges with the realization that many of eHsp90’s motogenic effectors are also facilitators of EMT, exemplified by TLR4 [231], EphA2 [232], EGFR [233,234,235], and MMPs [236,237]. Not surprisingly, these proteins exhibit dual roles in wound repair and tumor progression, a theme highlighting the conserved role of EMT in these processes. The relation between MMPs and EMT-TFs is complex. Although MMP activity may trigger EMT, EMT-TFs factors may also function as upstream regulators of MMPs, with their invasive function inextricably dependent upon MMP activity [238,239,240]. The ability of eHsp90 to activate MMPs also supports feed-forward mechanisms to drive EMT, as exemplified by the liberation of growth factors and activation of EGFR signaling [141,241] (and see Figure 2 and Figure 3). Moreover, eHsp90 promotes cell motility in cooperation with the intracellular effectors NF-kB, AKT, and ERK [10,76,121,141,201,216]. These effectors serve as molecular hubs for a large number of EMT-inducing signaling pathways [242] and may also drive EMT [243,244,245,246]. Our recent demonstration that ERK is essential for eHsp90-mediated EMT events [121] supports prior studies implicating ERK as an EMT activator [247,248,249,250], a function compatible with its known roles in development, wound healing and cancer [221,222,251].

## 8. Conclusions and Future Directions

The ability of eHsp90 to regulate inflammation, angiogenesis, matrix remodeling and cell motility is a perfect fit for its repair and regenerative properties. However, co-option of these activities in malignancy transforms eHsp90 into a powerful driver of tumor progression and metastasis. Tumor metastasis and wound healing share considerable phenotypic homology, including conservation of EMT activation. The ability of eHsp90 to regulate EMT events in wound healing and cancer offers considerable mechanistic insight into its orchestration of these processes. The secretion of Hsp90 from keratinocytes in wound healing or tumor cells in malignancy enables functional synergy between EMT-TFs and MMPs to facilitate remodeling and cell motility. While this transient response is useful for physiological wound closure and repair, these newly acquired properties endow cancer cells with the ability to invade and infiltrate the stroma, degrade the basement membrane, and ultimately access the vasculature to disseminate at distant sites. Interestingly, we reported that eHsp90 was highly secreted in more aggressive and mesenchymal prostate cell types [121], a pattern shared with breast cancers [87]. Placed within this current framework, these trends may indicate that eHsp90 expression plays a critical role in enforcing mesenchymal behavior. This interpretation is generally supported by clinical findings wherein patients with metastatic disease exhibited increased serum Hsp90 expression [87,124]. It is noteworthy that stimuli regulating Hsp90 secretion, such as hypoxia, growth factors, and oxidative stress are also known inducers of EMT events [225,237,252]. This supports the notion that nature designed Hsp90 secretion as a reinforcing strategy for protection and repair, in part by eliciting EMT activation. The bi-directional relationship between eHsp90-mediated effectors and EMT-TFs creates a toxic reinforcing alliance that undoubtedly supports the tumor microenvironment, and hypoxia, inflammation, and MMPs are well known collaborators in the metastatic cascade [160]. Thus, it will be important to define factors regulating Hsp90 secretion upon exposure to microenvironmental stressors commonly found in malignancy. 

Although targeting the broadly conserved pro-invasive functions of eHsp90 in malignancy is an appealing strategy, a more comprehensive understanding of eHsp90’s role within the context of tumor immunity is warranted. The necrotic or injury induced secretion of Hsp90 is designed to alert the immune system, to initiate a survey of the tissue damage and to elicit repair mechanisms. However, the majority of studies implicating eHsp90 as a supportive factor in tumor progression have utilized immunocompromised mice. As such, no studies have yet evaluated eHsp90 immunoregulatory functions in physiologically relevant settings, a shortcoming that limits our understanding of the potentially redeeming tumor-suppressive properties of eHsp90. Inflammation has the paradoxical property of both enabling and suppressing tumor development [253,254]. The ability of eHsp90 to functionally and/or physically partner with the inflammatory mediators LRP1, TLR4 and NF-kB supports the premise that eHsp90 is simply executing its evolutionary conserved role as a master regulator of inflammatory responses. While eHsp90 may possess intrinsic tumor-suppressive functions, the tumor microenvironment undoubtedly hijacks eHsp90’s pro-inflammatory functions, transforming eHsp90 into a major driver of tumor progression. Further studies are needed to understand the basis of this tipping point that converts eHsp90 into a malignant conspirator. Additionally, future work is required to define clear criteria for targeting the intracellular *vs*. the extracellular protein in cancers. Many chemotherapeutic drugs target signaling molecules and receptors essential for wound repair [227], further highlighting the shared molecular mechanisms in these processes. Now that eHsp90 is appreciated as a master regulator of tumor-supportive wound repair pathways, the rationale exists to explore clinically viable eHsp90-targeted approaches.

## References

[1] Frydman J. (2001). Folding of newly translated proteins *in vivo*: The role of molecular chaperones. Annu. Rev. Biochem..

[2] Taipale M., Jarosz D.F., Lindquist S. (2010). Hsp90 at the hub of protein homeostasis: Emerging mechanistic insights. Nat. Rev. Mol. Cell Biol..

[3] Whitesell L., Lindquist S.L. (2005). Hsp90 and the chaperoning of cancer. Nat. Rev. Cancer.

[4] Moulick K., Ahn J.H., Zong H., Rodina A., Cerchietti L., Gomes DaGama E.M., Caldas-Lopes E., Beebe K., Perna F., Hatzi K. (2011). Affinity-based proteomics reveal cancer-specific networks coordinated by hsp90. Nat. Chem. Biol..

[5] Lambert J.P., Ivosev G., Couzens A.L., Larsen B., Taipale M., Lin Z.Y., Zhong Q., Lindquist S., Vidal M., Aebersold R. (2013). Mapping differential interactomes by affinity purification coupled with data-independent mass spectrometry acquisition. Nat. Methods.

[6] Taipale M., Krykbaeva I., Koeva M., Kayatekin C., Westover K.D., Karras G.I., Lindquist S. (2012). Quantitative analysis of HSP90-client interactions reveals principles of substrate recognition. Cell.

[7] Webb C.P., Hose C.D., Koochekpour S., Jeffers M., Oskarsson M., Sausville E., Monks A., Vande Woude G.F. (2000). The geldanamycins are potent inhibitors of the hepatocyte growth factor/scatter factor-met-urokinase plasminogen activator-plasmin proteolytic network. Cancer Res..

[8] Xu W., Mimnaugh E., Rosser M.F., Nicchitta C., Marcu M., Yarden Y., Neckers L. (2001). Sensitivity of mature Erbb2 to geldanamycin is conferred by its kinase domain and is mediated by the chaperone protein Hsp90. J. Biol. Chem..

[9] Basso A.D., Solit D.B., Chiosis G., Giri B., Tsichlis P., Rosen N. (2002). Akt forms an intracellular complex with heat shock protein 90 (Hsp90) and Cdc37 and is destabilized by inhibitors of Hsp90 function. J. Biol. Chem..

[10] Gopal U., Bohonowych J.E., Lema-Tome C., Liu A., Garrett-Mayer E., Wang B., Isaacs J.S. (2011). A novel extracellular Hsp90 mediated co-receptor function for LRP1 regulates EphA2 dependent glioblastoma cell invasion. PLoS One.

[11] Breinig M., Mayer P., Harjung A., Goeppert B., Malz M., Penzel R., Neumann O., Hartmann A., Dienemann H., Giaccone G. (2011). Heat shock protein 90-sheltered overexpression of insulin-like growth factor 1 receptor contributes to malignancy of thymic epithelial tumors. Clin. Cancer Res..

[12] Picard D., Khursheed B., Garabedian M.J., Fortin M.G., Lindquist S., Yamamoto K.R. (1990). Reduced levels of Hsp90 compromise steroid receptor action *in vivo*. Nature.

[13] Isaacs J.S., Jung Y.J., Mimnaugh E.G., Martinez A., Cuttitta F., Neckers L.M. (2002). Hsp90 regulates a von hippel lindau-independent hypoxia-inducible factor-1 alpha-degradative pathway. J. Biol. Chem..

[14] Isaacs J.S., Xu W., Neckers L. (2003). Heat shock protein 90 as a molecular target for cancer therapeutics. Cancer Cell.

[15] Bohonowych J.E., Gopal U., Isaacs J.S. (2010). Hsp90 as a gatekeeper of tumor angiogenesis: Clinical promise and potential pitfalls. J. Oncol..

[16] Neckers L., Workman P. (2012). Hsp90 molecular chaperone inhibitors: Are we there yet?. Clin. Cancer Res..

[17] Luo W., Rodina A., Chiosis G. (2008). Heat shock protein 90: Translation from cancer to alzheimer’s disease treatment?. BMC Neurosci..

[18] Kang B.H., Plescia J., Dohi T., Rosa J., Doxsey S.J., Altieri D.C. (2007). Regulation of tumor cell mitochondrial homeostasis by an organelle-specific Hsp90 chaperone network. Cell.

[19] Chae Y.C., Caino M.C., Lisanti S., Ghosh J.C., Dohi T., Danial N.N., Villanueva J., Ferrero S., Vaira V., Santambrogio L. (2012). Control of tumor bioenergetics and survival stress signaling by mitochondrial Hsp90s. Cancer Cell.

[20] Radisky D.C., Stallings-Mann M., Hirai Y., Bissell M.J. (2009). Single proteins might have dual but related functions in intracellular and extracellular microenvironments. Nat. Rev. Mol. Cell Biol..

[21] Calderwood S.K., Mambula S.S., Gray P.J. (2007). Extracellular heat shock proteins in cell signaling and immunity. Ann. NY Acad. Sci..

[22] Butler G.S., Dean R.A., Smith D., Overall C.M. (2009). Membrane protease degradomics: Proteomic identification and quantification of cell surface protease substrates. Methods Mol. Biol..

[23] Weidle U.H., Maisel D., Klostermann S., Schiller C., Weiss E.H. (2011). Intracellular proteins displayed on the surface of tumor cells as targets for therapeutic intervention with antibody-related agents. Cancer Genomics Proteomics.

[24] Luo B., Lee A.S. (2013). The critical roles of endoplasmic reticulum chaperones and unfolded protein response in tumorigenesis and anticancer therapies. Oncogene.

[25] Hua Y., White-Gilbertson S., Kellner J., Rachidi S., Usmani S.Z., Chiosis G., Depinho R., Li Z., Liu B. (2013). Molecular chaperone gp96 is a novel therapeutic target of multiple myeloma. Clin. Cancer Res..

[26] Patel P.D., Yan P., Seidler P.M., Patel H.J., Sun W., Yang C., Que N.S., Taldone T., Finotti P., Stephani R.A. (2013). Paralog-selective Hsp90 inhibitors define tumor-specific regulation of HER2. Nat. Chem. Biol..

[27] Sherman M., Multhoff G. (2007). Heat shock proteins in cancer. Ann. NY Acad. Sci..

[28] Powers M.V., Clarke P.A., Workman P. (2008). Dual targeting of Hsc70 and Hsp72 inhibits Hsp90 function and induces tumor-specific apoptosis. Cancer Cell.

[29] Juhasz K., Lipp A.M., Nimmervoll B., Sonnleitner A., Hesse J., Haselgruebler T., Balogi Z. (2013). The complex function of Hsp70 in metastatic cancer. Cancers.

[30] Srivastava P.K., DeLeo A.B., Old L.J. (1986). Tumor rejection antigens of chemically induced sarcomas of inbred mice. Proc. Natl. Acad. Sci. USA.

[31] Udono H., Srivastava P.K. (1994). Comparison of tumor-specific immunogenicities of stress-induced proteins gp96, Hsp90, and Hsp70. J. Immunol..

[32] Suto R., Srivastava P.K. (1995). A mechanism for the specific immunogenicity of heat shock protein-chaperoned peptides. Science.

[33] Srivastava P.K., Menoret A., Basu S., Binder R.J., McQuade K.L. (1998). Heat shock proteins come of age: Primitive functions acquire new roles in an adaptive world. Immunity.

[34] Schild H., Rammensee H.G. (2000). Gp96—The immune system’s Swiss army knife. Nat. Immunol..

[35] Zheng H., Dai J., Stoilova D., Li Z. (2001). Cell surface targeting of heat shock protein gp96 induces dendritic cell maturation and antitumor immunity. J. Immunol..

[36] Nicchitta C.V., Carrick D.M., Baker-Lepain J.C. (2004). The messenger and the message: Gp96 (GRP94)-peptide interactions in cellular immunity. Cell Stress Chaperones.

[37] Flynn G.C., Chappell T.G., Rothman J.E. (1989). Peptide binding and release by proteins implicated as catalysts of protein assembly. Science.

[38] Arnold-Schild D., Hanau D., Spehner D., Schmid C., Rammensee H.G., de la Salle H., Schild H. (1999). Cutting edge: Receptor-mediated endocytosis of heat shock proteins by professional antigen-presenting cells. J. Immunol..

[39] Udono H., Srivastava P.K. (1993). Heat shock protein 70-associated peptides elicit specific cancer immunity. J. Exp. Med..

[40] Moroi Y., Mayhew M., Trcka J., Hoe M.H., Takechi Y., Hartl F.U., Rothman J.E., Houghton A.N. (2000). Induction of cellular immunity by immunization with novel hybrid peptides complexed to heat shock protein 70. Proc. Natl. Acad. Sci. USA.

[41] Massa C., Guiducci C., Arioli I., Parenza M., Colombo M.P., Melani C. (2004). Enhanced efficacy of tumor cell vaccines transfected with secretable hsp70. Cancer Res..

[42] Ostergaard L., Simonsen U., Eskildsen-Helmond Y., Vorum H., Uldbjerg N., Honore B., Mulvany M.J. (2009). Proteomics reveals lowering oxygen alters cytoskeletal and endoplasmatic stress proteins in human endothelial cells. Proteomics.

[43] Raiter A., Weiss C., Bechor Z., Ben-Dor I., Battler A., Kaplan B., Hardy B. (2010). Activation of GRP78 on endothelial cell membranes by an ADAM15-derived peptide induces angiogenesis. J. Vasc. Res..

[44] Zhang Y., Liu R., Ni M., Gill P., Lee A.S. (2010). Cell surface relocalization of the endoplasmic reticulum chaperone and unfolded protein response regulator GRP78/BiP. J. Biol. Chem..

[45] Ferrarini M., Heltai S., Zocchi M.R., Rugarli C. (1992). Unusual expression and localization of heat-shock proteins in human tumor cells. Int. J. Cancer..

[46] Tamura Y., Tsuboi N., Sato N., Kikuchi K. (1993). 70 kDa heat shock cognate protein is a transformation-associated antigen and a possible target for the host’s anti-tumor immunity. J. Immunol..

[47] Multhoff G., Botzler C., Wiesnet M., Muller E., Meier T., Wilmanns W., Issels R.D. (1995). A stress-inducible 72-kDa heat-shock protein (Hsp72) is expressed on the surface of human tumor cells, but not on normal cells. Int. J. Cancer.

[48] Berger C.L., Dong Z., Hanlon D., Bisaccia E., Edelson R.L. (1997). A lymphocyte cell surface heat shock protein homologous to the endoplasmic reticulum chaperone, immunoglobulin heavy chain binding protein bip. Int. J. Cancer.

[49] Misra U.K., Gonzalez-Gronow M., Gawdi G., Wang F., Pizzo S.V. (2004). A novel receptor function for the heat shock protein Grp78: Silencing of Grp78 gene expression attenuates alpha2M*-induced signalling. Cell. Signal..

[50] Arap M.A., Lahdenranta J., Mintz P.J., Hajitou A., Sarkis A.S., Arap W., Pasqualini R. (2004). Cell surface expression of the stress response chaperone GRP78 enables tumor targeting by circulating ligands. Cancer Cell.

[51] Graner M.W., Cumming R.I., Bigner D.D. (2007). The heat shock response and chaperones/heat shock proteins in brain tumors: Surface expression, release, and possible immune consequences. J. Neurosci..

[52] Gonzalez-Gronow M., Selim M.A., Papalas J., Pizzo S.V. (2009). GRP78: A multifunctional receptor on the cell surface. Antioxid. Redox Signal..

[53] Ni M., Zhang Y., Lee A.S. (2011). Beyond the endoplasmic reticulum: Atypical GRP78 in cell viability, signalling and therapeutic targeting. Biochem. J..

[54] Misra U.K., Deedwania R., Pizzo S.V. (2006). Activation and cross-talk between Akt, NF-κb, and unfolded protein response signaling in 1-LN prostate cancer cells consequent to ligation of cell surface-associated GRP78. J. Biol. Chem..

[55] Shani G., Fischer W.H., Justice N.J., Kelber J.A., Vale W., Gray P.C. (2008). GRP78 and cripto form a complex at the cell surface and collaborate to inhibit transforming growth factor beta signaling and enhance cell growth. Mol. Cell. Biol..

[56] Philippova M., Ivanov D., Joshi M.B., Kyriakakis E., Rupp K., Afonyushkin T., Bochkov V., Erne P., Resink T.J. (2008). Identification of proteins associating with glycosylphosphatidylinositol- anchored T-cadherin on the surface of vascular endothelial cells: Role for Grp78/BiP in T-cadherin-dependent cell survival. Mol. Cell. Biol..

[57] Kern J., Untergasser G., Zenzmaier C., Sarg B., Gastl G., Gunsilius E., Steurer M. (2009). GRP-78 secreted by tumor cells blocks the antiangiogenic activity of bortezomib. Blood.

[58] Asea A., Kraeft S.K., Kurt-Jones E.A., Stevenson M.A., Chen L.B., Finberg R.W., Koo G.C., Calderwood S.K. (2000). Hsp70 stimulates cytokine production through a CD14-dependant pathway, demonstrating its dual role as a chaperone and cytokine. Nat. Med..

[59] Vabulas R.M., Ahmad-Nejad P., Ghose S., Kirschning C.J., Issels R.D., Wagner H. (2002). Hsp70 as endogenous stimulus of the toll/interleukin-1 receptor signal pathway. J. Biol. Chem..

[60] Morales C., Rachidi S., Hong F., Sun S., Ouyang X., Wallace C., Zhang Y., Garret-Mayer E., Wu J., Liu B. (2014). Immune chaperone gp96 drives the contributions of macrophages to inflammatory colon tumorigenesis. Cancer Res..

[61] Gastpar R., Gehrmann M., Bausero M.A., Asea A., Gross C., Schroeder J.A., Multhoff G. (2005). Heat shock protein 70 surface-positive tumor exosomes stimulate migratory and cytolytic activity of natural killer cells. Cancer Res..

[62] Mambula S.S., Calderwood S.K. (2006). Heat shock protein 70 is secreted from tumor cells by a nonclassical pathway involving lysosomal endosomes. J. Immunol..

[63] Schmitt E., Gehrmann M., Brunet M., Multhoff G., Garrido C. (2007). Intracellular and extracellular functions of heat shock proteins: Repercussions in cancer therapy. J. Leukoc. Biol..

[64] Sims J.D., McCready J., Jay D.G. (2011). Extracellular heat shock protein (Hsp)70 and Hsp90α assist in matrix metalloproteinase-2 activation and breast cancer cell migration and invasion. PLoS One.

[65] Ullrich S.J., Robinson E.A., Law L.W., Willingham M., Appella E. (1986). A mouse tumor-specific transplantation antigen is a heat shock-related protein. Proc. Natl. Acad. Sci. USA.

[66] Srivastava P. (2002). Roles of heat-shock proteins in innate and adaptive immunity. Nat. Rev. Immunol..

[67] Kurotaki T., Tamura Y., Ueda G., Oura J., Kutomi G., Hirohashi Y., Sahara H., Torigoe T., Hiratsuka H., Sunakawa H. (2007). Efficient cross-presentation by heat shock protein 90-peptide complex-loaded dendritic cells via an endosomal pathway. J. Immunol..

[68] Murshid A., Gong J., Calderwood S.K. (2010). Heat shock protein 90 mediates efficient antigen cross presentation through the scavenger receptor expressed by endothelial cells-I. J. Immunol..

[69] Oura J., Tamura Y., Kamiguchi K., Kutomi G., Sahara H., Torigoe T., Himi T., Sato N. (2011). Extracellular heat shock protein 90 plays a role in translocating chaperoned antigen from endosome to proteasome for generating antigenic peptide to be cross-presented by dendritic cells. Int. Immunol..

[70] Basu S., Binder R.J., Ramalingam T., Srivastava P.K. (2001). CD91 is a common receptor for heat shock proteins gp96, Hsp90, Hsp70, and calreticulin. Immunity.

[71] Binder R.J., Vatner R., Srivastava P. (2004). The heat-shock protein receptors: Some answers and more questions. Tissue Antigens.

[72] El Hamidieh A., Grammatikakis N., Patsavoudi E. (2012). Cell surface Cdc37 participates in extracellular Hsp90 mediated cancer cell invasion. PLoS One.

[73] Song X., Wang X., Zhuo W., Shi H., Feng D., Sun Y., Liang Y., Fu Y., Zhou D., Luo Y. (2010). The regulatory mechanism of extracellular Hsp90α on matrix metalloproteinase-2 processing and tumor angiogenesis. J. Biol. Chem..

[74] Cheng C.F., Sahu D., Tsen F., Zhao Z., Fan J., Kim R., Wang X., O’Brien K., Li Y., Kuang Y. (2011). A fragment of secreted hsp90α carries properties that enable it to accelerate effectively both acute and diabetic wound healing in mice. J. Clin. Investig..

[75] Sahu D., Zhao Z., Tsen F., Cheng C.F., Park R., Situ A.J., Dai J., Eginli A., Shams S., Chen M. (2012). A potentially common peptide target in secreted heat shock protein-90alpha for hypoxia-inducible factor-1alpha-positive tumors. Mol. Biol. Cell.

[76] Bohonowych J., Hance M., Nolan K., Defee M., Parsons C., Isaacs J. (2014). Extracellular Hsp90 mediates an NF-κb dependent inflammatory stromal program: Implications for the prostate tumor microenvironment. Prostate.

[77] Basu S., Binder R.J., Suto R., Anderson K.M., Srivastava P.K. (2000). Necrotic but not apoptotic cell death releases heat shock proteins, which deliver a partial maturation signal to dendritic cells and activate the NF-κb pathway. Int. Immunol..

[78] Clayton A., Turkes A., Navabi H., Mason M.D., Tabi Z. (2005). Induction of heat shock proteins in B-cell exosomes. J. Cell Sci..

[79] Yu X., Harris S.L., Levine A.J. (2006). The regulation of exosome secretion: A novel function of the p53 protein. Cancer Res..

[80] Cheng C.F., Fan J., Fedesco M., Guan S., Li Y., Bandyopadhyay B., Bright A.M., Yerushalmi D., Liang M., Chen M. (2008). Transforming growth factor α (TGFα)-stimulated secretion of Hsp90α: Using the receptor LRP-1/CD91 to promote human skin cell migration against a TGFβ-rich environment during wound healing. Mol. Cell. Biol..

[81] McCready J., Sims J.D., Chan D., Jay D.G. (2010). Secretion of extracellular Hsp90alpha via exosomes increases cancer cell motility: A role for plasminogen activation. BMC Cancer.

[82] Ramteke A., Ting H., Agarwal C., Mateen S., Somasagara R., Hussain A., Graner M., Frederick B., Agarwal R., Deep G. (2013). Exosomes secreted under hypoxia enhance invasiveness and stemness of prostate cancer cells by targeting adherens junction molecules. Mol. Carcinog..

[83] Liao D.F., Jin Z.G., Baas A.S., Daum G., Gygi S.P., Aebersold R., Berk B.C. (2000). Purification and identification of secreted oxidative stress-induced factors from vascular smooth muscle cells. J. Biol. Chem..

[84] Yang Y., Rao R., Shen J., Tang Y., Fiskus W., Nechtman J., Atadja P., Bhalla K. (2008). Role of acetylation and extracellular location of heat shock protein 90α in tumor cell invasion. Cancer Res..

[85] Lv L.H., Wan Y.L., Lin Y., Zhang W., Yang M., Li G.L., Lin H.M., Shang C.Z., Chen Y.J., Min J. (2012). Anticancer drugs cause release of exosomes with heat shock proteins from human hepatocellular carcinoma cells that elicit effective natural killer cell antitumor responses *in vitro*. J. Biol. Chem..

[86] Lei H., Venkatakrishnan A., Yu S., Kazlauskas A. (2007). Protein kinase A-dependent translocation of Hsp90α impairs endothelial nitric-oxide synthase activity in high glucose and diabetes. J. Biol. Chem..

[87] Wang X., Song X., Zhuo W., Fu Y., Shi H., Liang Y., Tong M., Chang G., Luo Y. (2009). The regulatory mechanism of Hsp90α secretion and its function in tumor malignancy. Proc. Natl. Acad. Sci. USA.

[88] Song X., Luo Y. (2010). The regulatory mechanism of hsp90α secretion from endothelial cells and its role in angiogenesis during wound healing. Biochem. Biophys. Res. Commun..

[89] Li W., Li Y., Guan S., Fan J., Cheng C.F., Bright A.M., Chinn C., Chen M., Woodley D.T. (2007). Extracellular heat shock protein-90α: Linking hypoxia to skin cell motility and wound healing. EMBO J..

[90] Li W., Tsen F., Sahu D., Bhatia A., Chen M., Multhoff G., Woodley D.T. (2013). Extracellular Hsp90 (eHsp90) as the actual target in clinical trials: Intentionally or unintentionally. Int. Rev. Cell Mol. Biol..

[91] Thomaidou D., Patsavoudi E. (1993). Identification of a novel neuron-specific surface antigen in the developing nervous system, by monoclonal antibody 4C5. Neuroscience.

[92] Sidera K., Samiotaki M., Yfanti E., Panayotou G., Patsavoudi E. (2004). Involvement of cell surface Hsp90 in cell migration reveals a novel role in the developing nervous system. J. Biol. Chem..

[93] Thomaidou D., Yfanti E., Patsavoudi E. (1996). Expression of the 4C5 antigen during development and after injury of the rat sciatic nerve. J. Neurosci. Res..

[94] Yfanti E., Sidera K., Margaritis L.H., Patsavoudi E. (2004). The 4C5 antigen is associated with schwann cell migration during development and regeneration of the rat peripheral nervous system. Glia.

[95] Guenard V., Kleitman N., Morrissey T.K., Bunge R.P., Aebischer P. (1992). Syngeneic schwann cells derived from adult nerves seeded in semipermeable guidance channels enhance peripheral nerve regeneration. J. Neurosci..

[96] Thomaidou D., Dori I., Patsavoudi E. (1995). Developmental expression and functional characterization of the 4C5 antigen in the postnatal cerebellar cortex. J. Neurochem..

[97] Ishimoto T., Kamei A., Koyanagi S., Nishide N., Uyeda A., Kasai M., Taguchi T. (1998). Hsp90 has neurite-promoting activity *in vitro* for telencephalic and spinal neurons of chick embryos. Biochem. Biophys. Res. Commun..

[98] Sarkar A.A., Zohn I.E. (2012). Hectd1 regulates intracellular localization and secretion of Hsp90 to control cellular behavior of the cranial mesenchyme. J. Cell Biol..

[99] Martin P. (1997). Wound healing—Aiming for perfect skin regeneration. Science.

[100] Woodley D.T., Fan J., Cheng C.F., Li Y., Chen M., Bu G., Li W. (2009). Participation of the lipoprotein receptor LRP1 in hypoxia-Hsp90α autocrine signaling to promote keratinocyte migration. J. Cell Sci..

[101] Campana W.M., Li X., Dragojlovic N., Janes J., Gaultier A., Gonias S.L. (2006). The low-density lipoprotein receptor-related protein is a pro-survival receptor in schwann cells: Possible implications in peripheral nerve injury. J. Neurosci..

[102] Mantuano E., Jo M., Gonias S.L., Campana W.M. (2010). Low density lipoprotein receptor-related protein (LRP1) regulates Rac1 and Rhoa reciprocally to control schwann cell adhesion and migration. J. Biol. Chem..

[103] Gonias S.L., Campana W.M. (2014). LDL receptor-related protein-1: A regulator of inflammation in atherosclerosis, cancer, and injury to the nervous system. Am. J. Pathol..

[104] Herz J., Strickland D.K. (2001). Lrp: A multifunctional scavenger and signaling receptor. J. Clin. Investig..

[105] Mantuano E., Henry K., Yamauchi T., Hiramatsu N., Yamauchi K., Orita S., Takahashi K., Lin J.H., Gonias S.L., Campana W.M. (2011). The unfolded protein response is a major mechanism by which LRP1 regulates schwann cell survival after injury. J. Neurosci..

[106] Koong A.C., Denko N.C., Hudson K.M., Schindler C., Swiersz L., Koch C., Evans S., Ibrahim H., Le Q.T., Terris D.J. (2000). Candidate genes for the hypoxic tumor phenotype. Cancer Res..

[107] Montel V., Gaultier A., Lester R.D., Campana W.M., Gonias S.L. (2007). The low-density lipoprotein receptor-related protein regulates cancer cell survival and metastasis development. Cancer Res..

[108] Castellano J., Aledo R., Sendra J., Costales P., Juan-Babot O., Badimon L., Llorente-Cortes V. (2011). Hypoxia stimulates low-density lipoprotein receptor-related protein-1 expression through hypoxia-inducible factor-1alpha in human vascular smooth muscle cells. Arterioscler. Thromb. Vasc. Biol..

[109] Majno G., Gabbiani G., Hirschel B.J., Ryan G.B., Statkov P.R. (1971). Contraction of granulation tissue *in vitro*: Similarity to smooth muscle. Science.

[110] Vaughan M.B., Howard E.W., Tomasek J.J. (2000). Transforming growth factor-beta1 promotes the morphological and functional differentiation of the myofibroblast. Exp. Cell Res..

[111] Hinz B., Mastrangelo D., Iselin C.E., Chaponnier C., Gabbiani G. (2001). Mechanical tension controls granulation tissue contractile activity and myofibroblast differentiation. Am. J. Pathol..

[112] Hinz B., Phan S.H., Thannickal V.J., Prunotto M., Desmouliere A., Varga J., de Wever O., Mareel M., Gabbiani G. (2012). Recent developments in myofibroblast biology: Paradigms for connective tissue remodeling. Am. J. Pathol..

[113] Mirastschijski U., Haaksma C.J., Tomasek J.J., Agren M.S. (2004). Matrix metalloproteinase inhibitor GM 6001 attenuates keratinocyte migration, contraction and myofibroblast formation in skin wounds. Exp. Cell Res..

[114] Eustace B.K., Sakurai T., Stewart J.K., Yimlamai D., Unger C., Zehetmeier C., Lain B., Torella C., Henning S.W., Beste G. (2004). Functional proteomic screens reveal an essential extracellular role for Hsp90α in cancer cell invasiveness. Nat. Cell Biol..

[115] Stellas D., El Hamidieh A., Patsavoudi E. (2010). Monoclonal antibody 4C5 prevents activation of MMP2 and MMP9 by disrupting their interaction with extracellular HSP90 and inhibits formation of metastatic breast cancer cell deposits. BMC Cell Biol..

[116] Yamashita C.M., Dolgonos L., Zemans R.L., Young S.K., Robertson J., Briones N., Suzuki T., Campbell M.N., Gauldie J., Radisky D.C. (2011). Matrix metalloproteinase 3 is a mediator of pulmonary fibrosis. Am. J. Pathol..

[117] Correia A.L., Mori H., Chen E.I., Schmitt F.C., Bissell M.J. (2013). The hemopexin domain of MMP3 is responsible for mammary epithelial invasion and morphogenesis through extracellular interaction with Hsp90α. Genes Dev..

[118] Tsutsumi S., Scroggins B., Koga F., Lee M.J., Trepel J., Felts S., Carreras C., Neckers L. (2008). A small molecule cell-impermeant Hsp90 antagonist inhibits tumor cell motility and invasion. Oncogene.

[119] Luo L.Y., Herrera I., Soosaipillai A., Diamandis E.P. (2002). Identification of heat shock protein 90 and other proteins as tumour antigens by serological screening of an ovarian carcinoma expression library. Br. J. Cancer.

[120] Cid C., Regidor I., Poveda P.D., Alcazar A. (2009). Expression of heat shock protein 90 at the cell surface in human neuroblastoma cells. Cell Stress Chaperones.

[121] Hance M.W., Dole K., Gopal U., Bohonowych J.E., Jezierska-Drutel A., Neumann C.A., Liu H., Garraway I.P., Isaacs J.S. (2012). Secreted Hsp90 is a novel regulator of the epithelial to mesenchymal transition (EMT) in prostate cancer. J. Biol. Chem..

[122] Becker B., Multhoff G., Farkas B., Wild P.J., Landthaler M., Stolz W., Vogt T. (2004). Induction of Hsp90 protein expression in malignant melanomas and melanoma metastases. Exp. Dermatol..

[123] Hegmans J.P., Bard M.P., Hemmes A., Luider T.M., Kleijmeer M.J., Prins J.B., Zitvogel L., Burgers S.A., Hoogsteden H.C., Lambrecht B.N. (2004). Proteomic analysis of exosomes secreted by human mesothelioma cells. Am. J. Pathol..

[124] Burgess E.F., Ham A.J., Tabb D.L., Billheimer D., Roth B.J., Chang S.S., Cookson M.S., Hinton T.J., Cheek K.L., Hill S. (2008). Prostate cancer serum biomarker discovery through proteomic analysis of α-2 macroglobulin protein complexes. Proteomics Clin. Appl..

[125] Sun Y., Zang Z., Xu X., Zhang Z., Zhong L., Zan W., Zhao Y., Sun L. (2010). Differential proteomics identification of HSP90 as potential serum biomarker in hepatocellular carcinoma by two-dimensional electrophoresis and mass spectrometry. Int. J. Mol. Sci..

[126] Vidal C.I., Mintz P.J., Lu K., Ellis L.M., Manenti L., Giavazzi R., Gershenson D.M., Broaddus R., Liu J., Arap W. (2004). An Hsp90-mimic peptide revealed by fingerprinting the pool of antibodies from ovarian cancer patients. Oncogene.

[127] Conroy S.E., Sasieni P.D., Fentiman I., Latchman D.S. (1998). Autoantibodies to the 90 kDa heat shock protein and poor survival in breast cancer patients. Eur. J. Cancer.

[128] Trieb K., Gerth R., Holzer G., Grohs J.G., Berger P., Kotz R. (2000). Antibodies to heat shock protein 90 in osteosarcoma patients correlate with response to neoadjuvant chemotherapy. Br. J. Cancer.

[129] Chen J.S., Hsu Y.M., Chen C.C., Chen L.L., Lee C.C., Huang T.S. (2010). Secreted heat shock protein 90α induces colorectal cancer cell invasion through CD91/LRP-1 and NF-κb-mediated integrin alphaV expression. J. Biol. Chem..

[130] Hendrix A., Maynard D., Pauwels P., Braems G., Denys H., van den Broecke R., Lambert J., van Belle S., Cocquyt V., Gespach C. (2010). Effect of the secretory small GTPase Rab27b on breast cancer growth, invasion, and metastasis. J. Natl. Cancer Inst..

[131] Sidera K., Patsavoudi E. (2008). Extracellular Hsp90: Conquering the cell surface. Cell Cycle.

[132] Li W., Sahu D., Tsen F. (2012). Secreted heat shock protein-90 (Hsp90) in wound healing and cancer. Biochim. Biophys. Acta.

[133] Sidera K., Gaitanou M., Stellas D., Matsas R., Patsavoudi E. (2008). A critical role for Hsp90 in cancer cell invasion involves interaction with the extracellular domain of HER-2. J. Biol. Chem..

[134] Lagarrigue F., Dupuis-Coronas S., Ramel D., Delsol G., Tronchere H., Payrastre B., Gaits-Iacovoni F. (2010). Matrix metalloproteinase-9 is upregulated in nucleophosmin-anaplastic lymphoma kinase-positive anaplastic lymphomas and activated at the cell surface by the chaperone heat shock protein 90 to promote cell invasion. Cancer Res..

[135] Stellas D., Karameris A., Patsavoudi E. (2007). Monoclonal antibody 4C5 immunostains human melanomas and inhibits melanoma cell invasion and metastasis. Clin. Cancer Res..

[136] Fidler I.J. (2002). Critical determinants of metastasis. Semin. Cancer Biol..

[137] Overall C.M., Kleifeld O. (2006). Tumour microenvironment opinion: Validating matrix metalloproteinases as drug targets and anti-targets for cancer therapy. Nat. Rev. Cancer.

[138] Page-McCaw A., Ewald A.J., Werb Z. (2007). Matrix metalloproteinases and the regulation of tissue remodelling. Nat. Rev. Mol. Cell Biol..

[139] Stebbins C.E., Russo A.A., Schneider C., Rosen N., Hartl F.U., Pavletich N.P. (1997). Crystal structure of an Hsp90-geldanamycin complex: Targeting of a protein chaperone by an antitumor agent. Cell.

[140] Yarden Y., Sliwkowski M.X. (2001). Untangling the ErbB signalling network. Nat. Rev. Mol. Cell Biol..

[141] Thuringer D., Hammann A., Benikhlef N., Fourmaux E., Bouchot A., Wettstein G., Solary E., Garrido C. (2011). Transactivation of the epidermal growth factor receptor by heat shock protein 90 via toll-like receptor 4 contributes to the migration of glioblastoma cells. J. Biol. Chem..

[142] Wu S., Dole K., Hong F., Noman A.S., Issacs J., Liu B., Li Z. (2012). Chaperone gp96-independent inhibition of endotoxin response by chaperone-based peptide inhibitors. J. Biol. Chem..

[143] McCawley L.J., Matrisian L.M. (2001). Matrix metalloproteinases: They’re not just for matrix anymore!. Curr. Opin. Cell Biol..

[144] Alter N.M. (1925). Mechanical irritation as etiologic factor of cancer: Clinical observation. Am. J. Pathol..

[145] Dolberg D.S., Hollingsworth R., Hertle M., Bissell M.J. (1985). Wounding and its role in RSV-mediated tumor formation. Science.

[146] Takahashi H., Ogata H., Nishigaki R., Broide D.H., Karin M. (2010). Tobacco smoke promotes lung tumorigenesis by triggering IKKbeta- and JNK1-dependent inflammation. Cancer Cell.

[147] Ben-Neriah Y., Karin M. (2011). Inflammation meets cancer, with NF-κb as the matchmaker. Nat. Immunol..

[148] Hanahan D., Weinberg R.A. (2011). Hallmarks of cancer: The next generation. Cell.

[149] Singer A.J., Clark R.A. (1999). Cutaneous wound healing. N. Engl. J. Med..

[150] Gurtner G.C., Werner S., Barrandon Y., Longaker M.T. (2008). Wound repair and regeneration. Nature.

[151] Haddow A. (1972). Molecular repair, wound healing, and carcinogenesis: Tumor production a possible overhealing?. Ad. Cancer Res..

[152] Dvorak H.F. (1986). Tumors: Wounds that do not heal. Similarities between tumor stroma generation and wound healing. N. Engl. J. Med..

[153] Schauer I.G., Rowley D.R. (2011). The functional role of reactive stroma in benign prostatic hyperplasia. Differ. Res. Biol. Divers..

[154] Lokmic Z., Musyoka J., Hewitson T.D., Darby I.A. (2012). Hypoxia and hypoxia signaling in tissue repair and fibrosis. Int. Rev. Cell Mol. Biol..

[155] Folkman J. (1971). Tumor angiogenesis: Therapeutic implications. N. Engl. J. Med..

[156] Senger D.R., Galli S.J., Dvorak A.M., Perruzzi C.A., Harvey V.S., Dvorak H.F. (1983). Tumor cells secrete a vascular permeability factor that promotes accumulation of ascites fluid. Science.

[157] Shweiki D., Itin A., Soffer D., Keshet E. (1992). Vascular endothelial growth factor induced by hypoxia may mediate hypoxia-initiated angiogenesis. Nature.

[158] Chan D.A., Giaccia A.J. (2007). Hypoxia, gene expression, and metastasis. Cancer Metastasis Rev..

[159] Semenza G.L. (2012). Molecular mechanisms mediating metastasis of hypoxic breast cancer cells. Trends Mol. Med..

[160] Finger E.C., Giaccia A.J. (2010). Hypoxia, inflammation, and the tumor microenvironment in metastatic disease. Cancer Metastasis Rev..

[161] Eltzschig H.K., Carmeliet P. (2011). Hypoxia and inflammation. N. Engl. J. Med..

[162] Mamlouk S., Wielockx B. (2013). Hypoxia-inducible factors as key regulators of tumor inflammation. Int. J. Cancer.

[163] Kuraishy A., Karin M., Grivennikov S.I. (2011). Tumor promotion via injury- and death-induced inflammation. Immunity.

[164] Grivennikov S.I., Karin M. (2010). Inflammation and oncogenesis: A vicious connection. Curr. Opin. Genet. Dev..

[165] Mantovani A., Allavena P., Sica A., Balkwill F. (2008). Cancer-related inflammation. Nature.

[166] Li N., Grivennikov S.I., Karin M. (2011). The unholy trinity: Inflammation, cytokines, and STAT3 shape the cancer microenvironment. Cancer Cell.

[167] Hanahan D., Coussens L.M. (2012). Accessories to the crime: Functions of cells recruited to the tumor microenvironment. Cancer Cell.

[168] Barrientos S., Stojadinovic O., Golinko M.S., Brem H., Tomic-Canic M. (2008). Growth factors and cytokines in wound healing. Wound Repair Regen..

[169] Shaw T.J., Martin P. (2009). Wound repair at a glance. J. Cell Sci..

[170] Sandbo N., Dulin N. (2011). Actin cytoskeleton in myofibroblast differentiation: Ultrastructure defining form and driving function. Transl. Res..

[171] Martins V.L., Caley M., O’Toole E.A. (2013). Matrix metalloproteinases and epidermal wound repair. Cell Tissue Res..

[172] Kalluri R., Zeisberg M. (2006). Fibroblasts in cancer. Nat. Rev. Cancer.

[173] Hinz B., Phan S.H., Thannickal V.J., Galli A., Bochaton-Piallat M.L., Gabbiani G. (2007). The myofibroblast: One function, multiple origins. Am. J. Pathol..

[174] Dvorak H.F. (2003). Rous-whipple award lecture. How tumors make bad blood vessels and stroma. Am. J. Pathol..

[175] Orimo A., Gupta P.B., Sgroi D.C., Arenzana-Seisdedos F., Delaunay T., Naeem R., Carey V.J., Richardson A.L., Weinberg R.A. (2005). Stromal fibroblasts present in invasive human breast carcinomas promote tumor growth and angiogenesis through elevated SDF-1/CXCL12 secretion. Cell.

[176] Schafer M., Werner S. (2008). Cancer as an overhealing wound: An old hypothesis revisited. Nat. Rev. Mol. Cell Biol..

[177] Erez N., Truitt M., Olson P., Arron S.T., Hanahan D. (2010). Cancer-associated fibroblasts are activated in incipient neoplasia to orchestrate tumor-promoting inflammation in an NF-κb-dependent manner. Cancer Cell.

[178] Camps J.L., Chang S.M., Hsu T.C., Freeman M.R., Hong S.J., Zhau H.E., von Eschenbach A.C., Chung L.W. (1990). Fibroblast-mediated acceleration of human epithelial tumor growth *in vivo*. Proc. Natl. Acad. Sci. USA.

[179] Olumi A.F., Grossfeld G.D., Hayward S.W., Carroll P.R., Tlsty T.D., Cunha G.R. (1999). Carcinoma-associated fibroblasts direct tumor progression of initiated human prostatic epithelium. Cancer Res..

[180] Bhowmick N.A., Neilson E.G., Moses H.L. (2004). Stromal fibroblasts in cancer initiation and progression. Nature.

[181] Valastyan S., Weinberg R.A. (2011). Tumor metastasis: Molecular insights and evolving paradigms. Cell.

[182] Cirri P., Chiarugi P. (2012). Cancer-associated-fibroblasts and tumour cells: A diabolic liaison driving cancer progression. Cancer Metastasis Rev..

[183] Lund L.R., Green K.A., Stoop A.A., Ploug M., Almholt K., Lilla J., Nielsen B.S., Christensen I.J., Craik C.S., Werb Z. (2006). Plasminogen activation independent of UPA and TPA maintains wound healing in gene-deficient mice. EMBO J..

[184] Joyce J.A., Pollard J.W. (2009). Microenvironmental regulation of metastasis. Nat. Rev. Cancer.

[185] Nieto M.A. (2013). Epithelial plasticity: A common theme in embryonic and cancer cells. Science.

[186] Radisky D., Hagios C., Bissell M.J. (2001). Tumors are unique organs defined by abnormal signaling and context. Semin. Cancer Biol..

[187] Franco O.E., Shaw A.K., Strand D.W., Hayward S.W. (2010). Cancer associated fibroblasts in cancer pathogenesis. Semin. Cell Dev. Biol..

[188] Tuxhorn J.A., Ayala G.E., Smith M.J., Smith V.C., Dang T.D., Rowley D.R. (2002). Reactive stroma in human prostate cancer: Induction of myofibroblast phenotype and extracellular matrix remodeling. Clin. Cancer Res..

[189] Barron D.A., Rowley D.R. (2012). The reactive stroma microenvironment and prostate cancer progression. Endocr. Related Cancer.

[190] Saadi A., Shannon N.B., Lao-Sirieix P., O’Donovan M., Walker E., Clemons N.J., Hardwick J.S., Zhang C., Das M., Save V. (2010). Stromal genes discriminate preinvasive from invasive disease, predict outcome, and highlight inflammatory pathways in digestive cancers. Proc. Natl. Acad. Sci. USA.

[191] Chang H.Y., Sneddon J.B., Alizadeh A.A., Sood R., West R.B., Montgomery K., Chi J.T., van de Rijn M., Botstein D., Brown P.O. (2004). Gene expression signature of fibroblast serum response predicts human cancer progression: Similarities between tumors and wounds. PLoS Biol..

[192] Defee M.R., Qin Z., Dai L., Toole B.P., Isaacs J.S., Parsons C.H. (2011). Extracellular Hsp90 serves as a co-factor for NF-κb activation and cellular pathogenesis induced by an oncogenic herpesvirus. Am. J. Cancer Res..

[193] Triantafilou K., Triantafilou M., Dedrick R.L. (2001). A CD14-independent lps receptor cluster. Nat. Immunol..

[194] Qin S., Ni M., Wang X., Maurier-Mahe F., Shurland D.L., Rodrigues G.A. (2011). Inhibition of rpe cell sterile inflammatory responses and endotoxin-induced uveitis by a cell-impermeable Hsp90 inhibitor. Exp. Eye Res..

[195] Chung S.W., Lee J.H., Choi K.H., Park Y.C., Eo S.K., Rhim B.Y., Kim K. (2009). Extracellular heat shock protein 90 induces interleukin-8 in vascular smooth muscle cells. Biochem. Biophys. Res. Commun..

[196] Grivennikov S.I., Greten F.R., Karin M. (2010). Immunity, inflammation, and cancer. Cell.

[197] Kluwe J., Mencin A., Schwabe R.F. (2009). Toll-like receptors, wound healing, and carcinogenesis. J. Mol. Med..

[198] Rose W.A., Sakamoto K., Leifer C.A. (2012). TLR9 is important for protection against intestinal damage and for intestinal repair. Sci. Rep..

[199] Triantafilou M., Triantafilou K. (2004). Heat-shock protein 70 and heat-shock protein 90 associate with toll-like receptor 4 in response to bacterial lipopolysaccharide. Biochem. Soc. Trans..

[200] Basseres D.S., Baldwin A.S. (2006). Nuclear factor-kappab and inhibitor of kappab kinase pathways in oncogenic initiation and progression. Oncogene.

[201] Chen W.S., Chen C.C., Chen L.L., Lee C.C., Huang T.S. (2013). Secreted heat shock protein 90α (Hsp90α) induces nuclear factor-κb-mediated TCF12 protein expression to down-regulate E-cadherinand to enhance colorectal cancer cell migration and invasion. J. Biol. Chem..

[202] Hiratsuka S., Watanabe A., Sakurai Y., Akashi-Takamura S., Ishibashi S., Miyake K., Shibuya M., Akira S., Aburatani H., Maru Y. (2008). The S100A8-serum amyloid A3-TLR4 paracrine cascade establishes a pre-metastatic phase. Nat. Cell Biol..

[203] Gatti G., Quintar A.A., Andreani V., Nicola J.P., Maldonado C.A., Masini-Repiso A.M., Rivero V.E., Maccioni M. (2009). Expression of toll-like receptor 4 in the prostate gland and its association with the severity of prostate cancer. Prostate.

[204] Lillis A.P., van Duyn L.B., Murphy-Ullrich J.E., Strickland D.K. (2008). LDL receptor-related protein 1: Unique tissue-specific functions revealed by selective gene knockout studies. Physiol. Rev..

[205] Staudt N.D., Jo M., Hu J., Bristow J.M., Pizzo D.P., Gaultier A., VandenBerg S.R., Gonias S.L. (2013). Myeloid cell receptor LRP1/CD91 regulates monocyte recruitment and angiogenesis in tumors. Cancer Res..

[206] Ffrench-Constant C., van de Water L., Dvorak H.F., Hynes R.O. (1989). Reappearance of an embryonicpattern of fibronectin splicing during wound healing in the adult rat. J. Cell Biol..

[207] Clark R.A., Lanigan J.M., DellaPelle P., Manseau E., Dvorak H.F., Colvin R.B. (1982). Fibronectin and fibrin provide a provisional matrix for epidermal cell migration during wound reepithelialization. J. Investig. Dermatol..

[208] Stupack D.G., Cheresh D.A. (2002). ECM remodeling regulates angiogenesis: Endothelial integrins look for new ligands. Sci. STKE.

[209] Hunter M.C., O’Hagan K.L., Kenyon A., Dhanani K.C., Prinsloo E., Edkins A.L. (2014). Hsp90 binds directly to fibronectin (FN) and inhibition reduces the extracellular fibronectin matrix in breast cancer cells. PLoS One.

[210] Hynes R.O. (2009). The extracellular matrix: Not just pretty fibrils. Science.

[211] Liotta L.A., Tryggvason K., Garbisa S., Hart I., Foltz C.M., Shafie S. (1980). Metastatic potential correlates with enzymatic degradation of basement membrane collagen. Nature.

[212] Parks W.C., Wilson C.L., Lopez-Boado Y.S. (2004). Matrix metalloproteinases as modulators of inflammation and innate immunity. Nat. Rev. Immunol..

[213] Jodele S., Blavier L., Yoon J.M., DeClerck Y.A. (2006). Modifying the soil to affect the seed: Role of stromal-derived matrix metalloproteinases in cancer progression. Cancer Metastasis Rev..

[214] Lu P., Takai K., Weaver V.M., Werb Z. (2011). Extracellular matrix degradation and remodeling in development and disease. Cold Spring Harb. Perspect. Biol..

[215] Bissell M.J., Hines W.C. (2011). Why don’t we get more cancer? A proposed role of the microenvironment in restraining cancer progression. Nat. Med..

[216] Tsen F., Bhatia A., O’Brien K., Cheng C.F., Chen M., Hay N., Stiles B., Woodley D.T., Li W. (2013). Extracellular heat shock protein 90 signals through subdomain II and the NPVY motif of LRP-1 receptor to Akt1 and Akt2: A circuit essential for promoting skin cell migration *in vitro* and wound healing *in vivo*. Mol. Cell. Biol..

[217] Wykosky J., Debinski W. (2008). The EphA2 receptor and ephrinA1 ligand in solid tumors: Function and therapeutic targeting. Mol. Cancer Res. MCR.

[218] Vaught D., Brantley-Sieders D.M., Chen J. (2008). Eph receptors in breast cancer: Roles in tumor promotion and tumor suppression. Breast Cancer Res..

[219] Parrinello S., Napoli I., Ribeiro S., Wingfield Digby P., Fedorova M., Parkinson D.B., Doddrell R.D., Nakayama M., Adams R.H., Lloyd A.C. (2010). Ephb signaling directs peripheral nerve regeneration through sox2-dependent schwann cell sorting. Cell.

[220] Coulthard M.G., Morgan M., Woodruff T.M., Arumugam T.V., Taylor S.M., Carpenter T.C., Lackmann M., Boyd A.W. (2012). Eph/ephrin signaling in injury and inflammation. Am. J. Pathol..

[221] Ling S., Chang X., Schultz L., Lee T.K., Chaux A., Marchionni L., Netto G.J., Sidransky D., Berman D.M. (2011). An EGFR-ERK-SOX9 signaling cascade links urothelial development and regeneration to cancer. Cancer Res..

[222] Mace K.A., Pearson J.C., McGinnis W. (2005). An epidermal barrier wound repair pathway in *Drosophila* is mediated by grainy head. Science.

[223] Wong V.W., Rustad K.C., Akaishi S., Sorkin M., Glotzbach J.P., Januszyk M., Nelson E.R., Levi K., Paterno J., Vial I.N. (2012). Focal adhesion kinase links mechanical force to skin fibrosis via inflammatory signaling. Nat. Med..

[224] Chang L., Karin M. (2001). Mammalian map kinase signalling cascades. Nature.

[225] Polyak K., Weinberg R.A. (2009). Transitions between epithelial and mesenchymal states: Acquisition of malignant and stem cell traits. Nat. Rev. Cancer.

[226] Lim J., Thiery J.P. (2012). Epithelial-mesenchymal transitions: Insights from development. Development.

[227] Leopold P.L., Vincent J., Wang H. (2012). A comparison of epithelial-to-mesenchymal transition and re-epithelialization. Semin. Cancer Biol..

[228] Peinado H., Olmeda D., Cano A. (2007). Snail, Zeb and bHLH factors in tumour progression: An alliance against the epithelial phenotype?. Nat. Rev. Cancer.

[229] Chaffer C.L., Weinberg R.A. (2011). A perspective on cancer cell metastasis. Science.

[230] Zheng H., Kang Y. (2013). Multilayer control of the EMT master regulators. Oncogene.

[231] Jing Y.Y., Han Z.P., Sun K., Zhang S.S., Hou J., Liu Y., Li R., Gao L., Zhao X., Zhao Q.D. (2012). Toll-like receptor 4 signaling promotes epithelial-mesenchymal transition in human hepatocellular carcinoma induced by lipopolysaccharide. BMC Med..

[232] Huang J., Xiao D., Li G., Ma J., Chen P., Yuan W., Hou F., Ge J., Zhong M., Tang Y. (2013). Epha2 promotes epithelial-mesenchymal transition through the Wnt/beta-catenin pathway in gastric cancer cells. Oncogene.

[233] Barr S., Thomson S., Buck E., Russo S., Petti F., Sujka-Kwok I., Eyzaguirre A., Rosenfeld-Franklin M., Gibson N.W., Miglarese M. (2008). Bypassing cellular EGF receptor dependence through epithelial-to-mesenchymal-like transitions. Clin. Exp. Metastasis.

[234] Arnoux V., Nassour M., L’Helgoualc’h A., Hipskind R.A., Savagner P. (2008). Erk5 controls slug expression and keratinocyte activation during wound healing. Mol. Biol. Cell.

[235] Hardy K.M., Booth B.W., Hendrix M.J., Salomon D.S., Strizzi L. (2010). ErbB/EGF signaling and emt in mammary development and breast cancer. J. Mammary Gland Biol. Neoplasia.

[236] Radisky D.C., Levy D.D., Littlepage L.E., Liu H., Nelson C.M., Fata J.E., Leake D., Godden E.L., Albertson D.G., Nieto M.A. (2005). Rac1b and reactive oxygen species mediate MMP-3-induced EMT and genomic instability. Nature.

[237] Nistico P., Bissell M.J., Radisky D.C. (2012). Epithelial-mesenchymal transition: General principles and pathological relevance with special emphasis on the role of matrix metalloproteinases. Cold Spring Harb. Perspect. Biol..

[238] Ota I., Li X.Y., Hu Y., Weiss S.J. (2009). Induction of a MT1-MMP and MT2-MMP-dependent basement membrane transmigration program in cancer cells by snail1. Proc. Natl. Acad. Sci. USA.

[239] Eckert M.A., Lwin T.M., Chang A.T., Kim J., Danis E., Ohno-Machado L., Yang J. (2011). Twist1-induced invadopodia formation promotes tumor metastasis. Cancer Cell.

[240] Weiss M.B., Abel E.V., Mayberry M.M., Basile K.J., Berger A.C., Aplin A.E. (2012). Twist1 is an ERK1/2 effector that promotes invasion and regulates MMP-1 expression in human melanoma cells. Cancer Res..

[241] Lue H.W., Yang X., Wang R., Qian W., Xu R.Z., Lyles R., Osunkoya A.O., Zhou B.P., Vessella R.L., Zayzafoon M. (2011). LIV-1 promotes prostate cancer epithelial-to-mesenchymal transition and metastasis through HB-EGF shedding and egfr-mediated erk signaling. PLoS One.

[242] Thiery J.P., Sleeman J.P. (2006). Complex networks orchestrate epithelial-mesenchymal transitions. Nat. Rev. Mol. Cell Biol..

[243] Chua H.L., Bhat-Nakshatri P., Clare S.E., Morimiya A., Badve S., Nakshatri H. (2007). NF-κb represses E-cadherin expression and enhances epithelial to mesenchymal transition of mammary epithelial cells: Potential involvement of Zeb-1 and Zeb-2. Oncogene.

[244] Kim H.J., Litzenburger B.C., Cui X., Delgado D.A., Grabiner B.C., Lin X., Lewis M.T., Gottardis M.M., Wong T.W., Attar R.M. (2007). Constitutively active type I insulin-like growth factor receptor causes transformation and xenograft growth of immortalized mammary epithelial cells and is accompanied by an epithelial-to-mesenchymal transition mediated by NF-κb and snail. Mol. Cell. Biol..

[245] Min C., Eddy S.F., Sherr D.H., Sonenshein G.E. (2008). NF-κb and epithelial to mesenchymal transition of cancer. J. Cell. Biochem..

[246] Xue G., Restuccia D.F., Lan Q., Hynx D., Dirnhofer S., Hess D., Ruegg C., Hemmings B.A. (2012). AKT/PKB-mediated phosphorylation of twist1 promotes tumor metastasis via mediating cross-talk between PI3K/AKT and TGF-beta signaling axes. Cancer Discov..

[247] Shin S., Dimitri C.A., Yoon S.O., Dowdle W., Blenis J. (2010). ERK2 but not ERK1 induces epithelial-to-mesenchymal transformation via DEF motif-dependent signaling events. Mol. Cell.

[248] Shin S.Y., Rath O., Zebisch A., Choo S.M., Kolch W., Cho K.H. (2010). Functional roles of multiple feedback loops in extracellular signal-regulated kinase and Wnt signaling pathways that regulate epithelial-mesenchymal transition. Cancer Res..

[249] Caramel J., Papadogeorgakis E., Hill L., Browne G.J., Richard G., Wierinckx A., Saldanha G., Osborne J., Hutchinson P., Tse G. (2013). A switch in the expression of embryonic EMT-inducers drives the development of malignant melanoma. Cancer Cell.

[250] Buonato J.M., Lazzara M.J. (2014). ERK1/2 blockade prevents epithelial-mesenchymal transition in lung cancer cells and promotes their sensitivity to egfr inhibition. Cancer Res..

[251] Deschenes-Simard X., Kottakis F., Meloche S., Ferbeyre G. (2014). ERKs in cancer: Friends or foes?. Cancer Res..

[252] Yang M.H., Wu M.Z., Chiou S.H., Chen P.M., Chang S.Y., Liu C.J., Teng S.C., Wu K.J. (2008). Direct regulation of twist by HIF-1α promotes metastasis. Nat. Cell Biol..

[253] Zou W. (2005). Immunosuppressive networks in the tumour environment and their therapeutic relevance. Nat. Rev. Cancer.

[254] De Visser K.E., Eichten A., Coussens L.M. (2006). Paradoxical roles of the immune system during cancer development. Nat. Rev. Cancer.

